# Sensory Neuropathy Affects Cardiac miRNA Expression Network Targeting *IGF-1*, *SLC2a-12*, *EIF-4e*, and *ULK-2* mRNAs

**DOI:** 10.3390/ijms20040991

**Published:** 2019-02-25

**Authors:** Péter Bencsik, Krisztina Kiss, Bence Ágg, Júlia A. Baán, Gergely Ágoston, Albert Varga, Kamilla Gömöri, Luca Mendler, Nóra Faragó, Ágnes Zvara, Péter Sántha, László G. Puskás, Gábor Jancsó, Péter Ferdinandy

**Affiliations:** 1 Cardiovascular Research Group, Department of Biochemistry, University of Szeged, Dóm tér 9, H-6720 Szeged, Hungary; bencsik.peter@med.u-szeged.hu (P.B.); k.krisztina88@gmail.com (K.K.); 2 Pharmahungary Group, Graphisoft Park, Záhony utca 7, H-1031 Budapest, Hungary; bence.agg@pharmahungary.com (B.Á.); kamilla.gomori@pharmahungary.com (K.G.); 3 Department of Pharmacology and Pharmacotherapy, University of Szeged, Dóm tér 12, H-6720 Szeged, Hungary; 4 Department of Pharmacology and Pharmacotherapy, Semmelweis University, Nagyvárad tér 4, H-1085 Budapest, Hungary; 5 Heart and Vascular Center, Semmelweis University, Városmajor utca 68, H-1122 Budapest, Hungary; 6 Muscle Adaptation Group, Department of Biochemistry, University of Szeged, Dóm tér 9, H-6720 Szeged, Hungary; baanjulia@gmail.com (J.A.B.); mendler.luca@med.u-szeged.hu (L.M.); 7 Institute of Family Medicine, University of Szeged, Tisza Lajos krt. 109., H-6720 Szeged, Hungary; agoston.gergely@med.u-szeged.hu (G.Á.); varga.albert@med.u-szeged.hu (A.V.); 8 Institute of Biochemistry II, Goethe University Medical School, University Hospital Building 75, Theodor-Stern-Kai 7, 60590 Frankfurt am Main, Germany; 9 Institute of Genetics, Biological Research Center, Hungarian Academy of Sciences, Temesvári körút 62, H-6726 Szeged, Hungary; nora@avidinbiotech.com (N.F.); zvara.agnes@gmail.com (Á.Z.); laszlo@avidinbiotech.com (L.G.P.); 10 Department of Physiology, University of Szeged, Dóm tér 10, H-6720 Szeged, Hungary; santha.peter@med.u-szeged.hu (P.S.); jancso.gabor@med.u-szeged.hu (G.J.)

**Keywords:** capsaicin, heart, network analysis, microRNA, sensory neuropathy

## Abstract

Background: Here we examined myocardial microRNA (miRNA) expression profile in a sensory neuropathy model with cardiac diastolic dysfunction and aimed to identify key mRNA molecular targets of the differentially expressed miRNAs that may contribute to cardiac dysfunction. Methods: Male Wistar rats were treated with vehicle or capsaicin for 3 days to induce systemic sensory neuropathy. Seven days later, diastolic dysfunction was detected by echocardiography, and miRNAs were isolated from the whole ventricles. Results: Out of 711 known miRNAs measured by miRNA microarray, the expression of 257 miRNAs was detected in the heart. As compared to vehicle-treated hearts, *miR-344b*, *miR-466b*, *miR-98*, *let-7a*, *miR-1*, *miR-206*, and *miR-34b* were downregulated, while *miR-181a* was upregulated as validated also by quantitative real time polymerase chain reaction (qRT-PCR). By an in silico network analysis, we identified common mRNA targets (insulin-like growth factor 1 (*IGF-1*), solute carrier family 2 facilitated glucose transporter member 12 (*SLC2a-12*), eukaryotic translation initiation factor 4e *(EIF-4e*), and Unc-51 like autophagy activating kinase 2 (*ULK-2*)) targeted by at least three altered miRNAs. Predicted upregulation of these mRNA targets were validated by qRT-PCR. Conclusion: This is the first demonstration that sensory neuropathy affects cardiac miRNA expression network targeting *IGF-1*, *SLC2a-12*, *EIF-4e*, and *ULK-2*, which may contribute to cardiac diastolic dysfunction. These results further support the need for unbiased omics approach followed by in silico prediction and validation of molecular targets to reveal novel pathomechanisms.

## 1. Introduction

Polyneuropathy including sensory neuropathy is one of the most common long-term complication of diabetes mellitus [1], which occurs in more than 50% of patients with long-standing diabetes [2]; for a review, see [3]. Diabetes is a well-established comorbidity of cardiovascular diseases including acute myocardial infarction (AMI) and heart failure (HF). Diabetic sensory neuropathy [4], see [3] for a review, affects the heart due to its rich sensory innervations [1,5].

Systemic administration of appropriate dose of capsaicin, a highly selective sensory neurotoxin, leads to a selective dysfunction of a morphologically well-defined group of primary sensory nerves [6,7,8]. Therefore, sensory desensitization induced by systemic capsaicin treatment is a well-accepted model to investigate the pathology and pharmacology of sensory neuropathy, see [9,10] for reviews. Cardiac sensory nerves play a pivotal role in myocardial adaptation processes to ischemic injury including ischemic pre-, post-, and remote conditioning [11,12,13]. We have previously shown that capsaicin-induced sensory neuropathy has led to cardiac diastolic dysfunction characterized by increased left ventricular end-diastolic pressure via different pathways dependent on nitric oxide and sarcoplasmic reticulum calcium ATPase [11,14,15]. Furthermore, capsaicin-induced sensory neuropathy has affected the expression profile of cardiac mRNAs [16]. Therefore, it was feasible to speculate that systemic sensory desensitization may also affect microRNA expression in the heart.

MicroRNAs (miRNAs) are 17–25 nucleotide-long noncoding RNAs inhibiting gene expression at posttranscriptional level via blocking of translation or enhanced degradation of messenger RNAs (mRNA) in a tissue specific manner, see [17,18,19] for reviews. To date, the number of identified miRNAs exceeds 35,000 in over 200 different species (http://www.mirbase.org). miRNAs are able to target several mRNAs and most mRNAs have different binding sites for multiple miRNAs; see [17] for a review. Many miRNAs have been identified in cardiovascular physiology or pathologies such as coronary artery stenosis, acute coronary syndrome, acute myocardial infarction, unstable angina, hypertension, heart failure, cardiomyopathies, etc., see [17,18,19] for reviews. Moreover, several altered miRNAs have been identified in the heart of diabetic animals or patients (see [20] for a review) and in diabetic complications (see [21] for a review) including diabetic neuropathy and sensory desensitization; see [3] for a review.

To identify molecular targets involved in disease pathomechanisms, the use of unbiased omics approach followed by in silico analysis of molecular networks are emerging, see [22] for a recent position paper. MiRNA omics followed by in silico target prediction and experimental validation of molecular targets are useful unbiased workflow for the identification of novel pathomechanisms and potential molecular targets [22,23,24]. Therefore, the aim of the present study was to identify novel mechanisms involved in cardiac diastolic dysfunction induced by sensory neuropathy by an unbiased workflow according to the recent recommendations of the European Society of Cardiology, Working Group of Cellular Biology of the Heart [22]. Accordingly, here we measured the cardiac miRNA expression profile followed by in silico miRNA–mRNA target interaction network analysis and experimental validation of predicted mRNA targets in a rat model of capsaicin-induced sensory neuropathy.

## 2. Results

### 2.1. Basic Characteristics

Body and heart weights were measured at baseline and at the 10th day, 7 days after the last capsaicin dose. Body weight and heart weight were significantly lower at the 10th day in sensory neuropathic group as compared to the vehicle-treated control group (Table 1). The sensory neuropathic animals gained significantly less weight as compared to the control animals (Table 1). The development of sensory neuropathy was confirmed in all animals treated with capsaicin by eye-wipe behavioral test. No mortality occurred either during capsaicin or vehicle treatment or during the 7-day follow-up.

### 2.2. Myocardial Function

Capsaicin-induced sensory neuropathy significantly altered functional parameters in the heart compared to vehicle-treated group as assessed by transthoracic echocardiography. Among other parameters (see Table 2) in sensory neuropathic hearts, end-diastolic diameter (EDD; for representative M-mode images, see Supplementary Figure S1), interventricular septum thickness (IVS), stroke volume (SV), as well as mitral valve velocity time index (MVVTI) were significantly decreased as compared to the vehicle-treated control group. Furthermore, Aa/Ea ratio showed as light decreasing tendency in sensory neuropathy group. Systolic performance of the heart did not show any difference between the two groups.

### 2.3. miRNA Microarray Analysis

To detect the alteration in miRNA expression profile of sensory neuropathic hearts, we performed miRNA microarray. Out of the 711 known miRNAs, the expression of 257 miRNAs was detectable. Out of the detected 257 miRNAs, *miR-344b* and *miR-466b* showed significant downregulation and *miR-181a* was upregulated as compared to the vehicle-treated control (Table 3). Despite *miR-98*, *let-7a*, *miR-1*, and *miR-206* as well as *miR-34b* not showing significant changes in sensory neuropathic animals as compared to controls, we selected these miRNAs for further analyses since their log_2_ ratios were <−0.6 and >0.6, respectively (Table 3).

### 2.4. Validation of miRNA Microarray Results by qRT-PCR

In order to validate miRNA microarray analysis, we used qRT-PCR (Table 4). Significant downregulation of *miR-466b*, *miR-98*, *let-7a*, *miR-1*, and *miR-206* as well as upregulation of *miR-181a* were confirmed by qRT-PCR (Table 4). *miR-34b* showed a significant downregulation by qRT-PCR (Table 4); however, it showed a non-significant upregulation in the microarray (Table 3). The expression of *miR-344b* could not be detected by qRT-PCR (Table 4). The expression changes of 6 out of 8 selected microRNAs were confirmed by qRT-PCR, which shows an acceptable rate of confirmation of the microarray data [25,26,27,28].

### 2.5. In Silico Network Analysis

**Figure 1:**
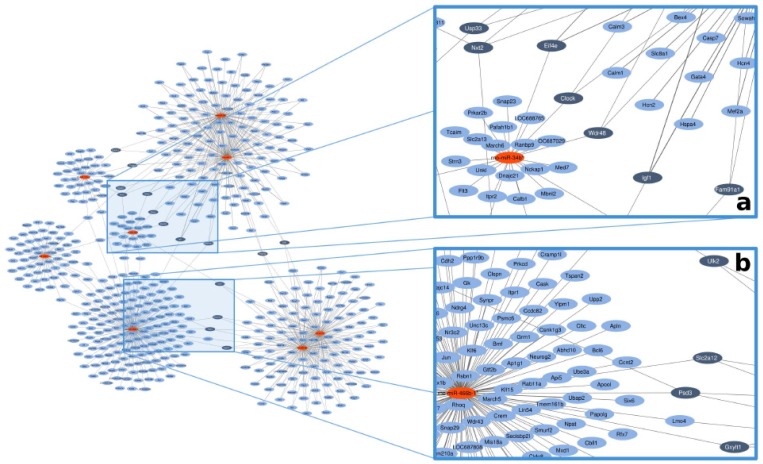
Representative image for in silico network analysis of the possible gene targets of the 8 altered miRNAs based on online databanks. Red nodes present miRNAs, blue nodes mark the predicted targets. Dark blue nodes label the target genes with ≥3 miRNA connections. Edges (gray lines) between nodes represent predicted miRNA–target interactions. (**a**) and (**b**) panels are presented in a magnified manner to help better perceive them.

In order to determine targets of the altered miRNAs, we used a previously validated software [23] relying on 3 publicly available online databases and illustrated the results on a miRNA–target network. We identified 15 target genes with high miRNA connectivity (≥degree 3) (Figure 1, dark blue spots). Out of 15 genes, we selected 4 targets based on available literature related to myocardial function and/or diabetes. Insulin-like growth factor-1 (*IGF-1*) was regulated by *miR-466b*, *miR-1*, and *miR-206*; solute carrier family 2 facilitated glucose transporter member 12 (*SLC2a-12*) was regulated by *miR-466b*, *miR-98*, and *let-7a*; eukaryotic translation initiation factor 4e (*EIF-4e*) was regulated by *miR-1*, *miR-206*, and *miR-34b*; and Unc-51 like autophagy activating kinase 2 (*ULK-2*) was regulated by *miR-98*, *let-7a*, and *miR-34b* (Figure 1, Table 5). Each of these miRNAs were downregulated by capsaicin treatment in the heart. For more miRNA–target connections, see Table S1.

### 2.6. Validation of Gene Targets at mRNA Level

**Figure 2:**
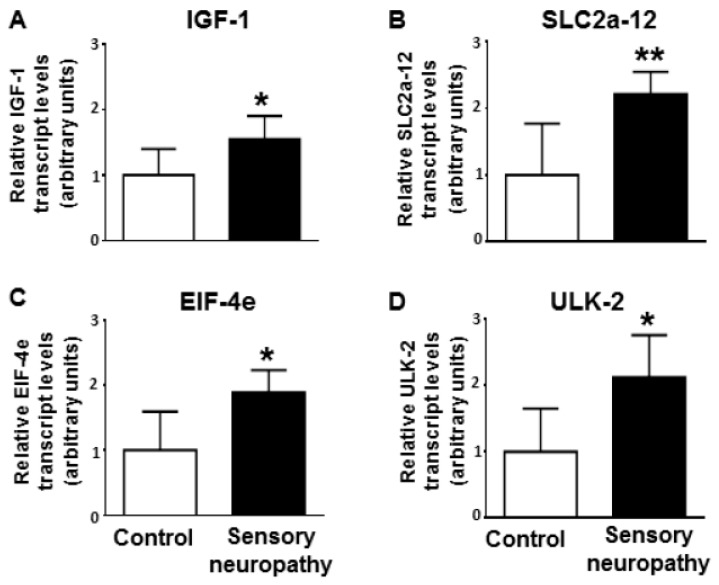
mRNA levels of (**A**) *IGF-1* (insulin-like growth factor 1), (**B**) *SLC2a-12* (solute carrier family 2 facilitated glucose transporter member 12), (**C**) *EIF-4e* (eukaryotic translation initiation factor 4e), and (**D**) *ULK-2* (Unc-51 like autophagy activating kinase 2) in sensory neuropathic rat heart samples as compared to vehicle-treated controls (control). The transcript levels were normalized to *GAPDH* (glyceraldehyde-3-phosphate dehydrogenase). Data are expressed in arbitrary units as means ± SD. (n = 5–6, * *p* < 0.05, ** *p* < 0.01 versus control; unpaired Student’s t-test).

We investigated the mRNA level of the 4 selected gene targets by qRT-PCR to validate the results of microarray and in silico network analysis (a representative agarose gel image of amplified cDNA transcript is available in Supplementary Figure S2). The levels of mRNAs were significantly elevated in the case of *IGF-1*, *SLC2a-12*, *EIF-4e*, and *ULK-2* in the sensory neuropathic group as compared to the controls (Figure 2 A–D). We used *GAPDH* for internal control, because GAPDH levels were similar in both control and sensory neuropathic heart samples (Figure S3).

## 3. Discussion

Here, we have shown that sensory neuropathy induced by systemic capsaicin treatment leads to the downregulation of 7 miRNAs and to the upregulation of 1 miRNA in the rat heart. Based on the alteration of these miRNAs, in silico miRNA–mRNA target network analysis predicted changes in the expression of *IGF-1*, *SLC2a-12*, *EIF-4e*, and *ULK-2* genes, which was then validated by qRT-PCR in the heart. This is the first demonstration that sensory neuropathy affects cardiac miRNA expression network targeting *IGF-1*, *SLC2a-12*, *EIF-4e*, and *ULK-2*, which may contribute to cardiac diastolic dysfunction induced by sensory neuropathy. These results further support the need for unbiased omics approach followed by in silico prediction and validation molecular targets to reveal novel pathomechanisms according to the recent recommendations of the European Society of Cardiology Working Group of Cellular Biology of the Heart [22].

### 3.1. Altered Cardiac Function Due to Sensory Neuropathy

Here, we found that in vivo cardiac diastolic function was impaired by capsaicin desensitization, which supports our previous ex vivo functional data [11,14]. End-diastolic diameter and interventricular septum thickness as well as stroke volume and mitral valve velocity time index were significantly decreased as compared to the vehicle-treated control group. Furthermore, Aa/Ea ratio showed a slight decreasing tendency in sensory neuropathy group, which indicated cardiac diastolic dysfunction according to previously published data [29].

The physiological and pathophysiological roles of sensory nerves in cardiac function in the healthy or diseased heart is somewhat neglected in the literature, therefore, little is known about it. However, some mechanisms have been described, including, e.g., regulation of cardiac contractility by different neuropeptide and nitric oxide-mediated mechanisms [14,16,30,31,32,33,34,35]. Therefore, here we used an unbiased non-hypothesis driven miRNA transcriptomics-based workflow to identify novel molecular targets that may be involved in the mechanisms of cardiac dysfunction caused by sensory neuropathy.

### 3.2. Altered Cardiac miRNAs Expression Profile due to Sensory Neuropathy

By using the unbiased omics approach to reveal global cardiac miRNA expression changes, we have shown the downregulation of *miR-344b*, *miR-466b*, *miR-98*, *let-7a*, *miR-1*, *miR-206*, *miR-34b*, and an upregulation of *miR-181a* in the heart in a rat model of sensory neuropathy induced by systemic capsaicin treatment. Regarding *miR-1* and *miR-206* downregulation in the heart, concordant results were shown in the soleus muscle after sciatic nerve denervation [36,37]. *miR-1* and *miR-206* belong to “myomir” network having a potent role in cardiac and skeletal muscle development, regulation, and remodeling, see [17,38,39] for reviews. Previously, we have found an increase in superoxide dismutase-1 (SOD1) and a decrease in endothelial nitrogen monoxide synthase (eNOS) activities as well as a reduction of nitrogen monoxide (NO) levels in hearts of sensory desensitized rats [11,14,15]. In line with this, *SOD1* expression was repressed in dog hearts overexpressing *miR-206* [40], and the protein level of SOD1 was decreased under oxidative conditions in *miR-1* overexpressing mice [41]. Moreover, in this sensory neuropathy model, we have previously shown that cardiac level of S-nitrosylated SERCA2a was decreased [14]. Other studies showed connection between *SERCA2a* and *miR-1* in the heart [42,43]: *miR-1* was downregulated in post-infarction heart failure in rats, and its expression was normalized via reversed remodeling by adenoviral *SERCA2a* gene delivery [42]. In different diabetic conditions, the alteration of *miR-1* and *miR-206* seems to be less clear. Cardiac *miR-1* and *miR-206* were downregulated 2 months (mice) or 5 weeks (rats) after induction of diabetes by streptozotocin (STZ) treatment [44,45], and *miR-206* was downregulated in vascular smooth muscle cells in hyperglycemic conditions [46]. In contrast, others found that both miRNAs were upregulated in hearts of STZ-treated rats 2 weeks after STZ injection [47]. However, in these diabetic models sensory neuropathy was not investigated. Further studies proved that downregulation of *miR-1* contributes to pathological cardiac hypertrophy including diabetic cardiomyopathy, see [38,39] for reviews, but others found upregulation in human cardiomyopathies or heart failure [48]. Moreover, *miR-1* expression was also increased in AMI patients [49,50], as well as in rodents during ischemic pre- or postconditioning [26,51].

*MiR-98* and *let-7a* were downregulated in the present capsaicin-induced sensory neuropathy model. Both miRNAs belong to the let-7 family, see [52] for a review. Bao et al. have found that *let-7a* overexpression resulted in an increase in *eNOS* expression and NO content in endothelial cells [53], which conforms to our previous and present findings in sensory neuropathic animals, i.e., *let-7a* was downregulated as well as *eNOS* expression and NO content were decreased in the heart [14]. Moreover, previous studies showed that *let-7a* were downregulated in the hearts of diabetic mice [54] as well as in the serum of diabetic patients [55] or in heart samples of rodent AMI models [26,56,57]. Only a few publications are available for investigating *miR-98* in connection with sensory neuropathy or cardiac pathologies. Downregulation of *miR-98* was shown to lead to the upregulation of *cyclin D2*, which is involved in the vascular complications of type 2 diabetes mellitus [58,59], and upregulation of *miR-98* prevented cardiac hypertrophy via inhibition of *cyclin D2* [60]. Here, we also identified cyclin D2 (*Ccnd2*) as a possible target for *miR-98* (Figure 1).

We found here that *miR-34b* was repressed in sensory neuropathy. There is no available literature about the alteration of *miR-34b* in neuropathic models. Previous studies described upregulation of *miR-34b* in hearts of patients with diabetes or with end-stage heart failure [61,62]. Inhibition of miR-34 family (*miR-34a*, *-34b*, and *-34c*) by an anti-miR improved cardiac function and prevented remodeling in mice after AMI or in pressure overload-induced cardiac hypertrophy [63]. Therefore, *mir-34b* and its targets may be involved in the diastolic dysfunction of the heart observed in the present study.

Although we found repression of *miR-466b* and *miR-344b* in sensory neuropathy model, there is a gap in the literature investigating the role of these miRNAs in the heart or in diabetes or in neuropathy models. A previous study showed that *miR-466b* is downregulated in neuronal stem cells isolated from embryos of hyperglycemic pregnant mice [64]. Liu et al. have previously identified *miR-344b* in the developing mouse brain, but they have not found *miR-344b* in the embryonic mouse heart [65]. Based on the above-mentioned previous findings, we can hypothesize that *miR-466b* and *miR-344b* may rather be derived from cardiac neural cells than from cardiomyocytes.

*miR-181a* was the only miRNA, which showed significant upregulation in our present study. *miR-181a* was downregulated in exercise-induced cardiac hypertrophy in rats [66]. However, *miR-181* was upregulated in the serum of diabetic patients [67,68] and during acute ischemia/reperfusion injury or in ischemic preconditioning in rats [26]. We have previously found that myocardial ROS production including NO and peroxynitrite levels were decreased in capsaicin-induced neuropathy model [14], which may be related to the roles of NO and peroxynitrite in myocardial ischemia or in cardiac preconditioning. Thus, it seems that *miR-181a* contributes to cardiac pathologies—it is upregulated during acute injuries and repressed in chronic disease states.

### 3.3. In Silico miRNA–mRNA Target Prediction

In the present study, we successfully applied and further validated the recently developed miRNAtarget™ software [23] to identify the possible targets of the altered miRNAs according to our results of experimental validation of predicted targets (see Section 4.4.). Several different sources of possible miRNA–target gene interactions (MTIs) on various level of maturity are available publicly; however, none of them provide a comprehensive solution for finding all existing MTIs for any randomly selected miRNA. While experimentally validated and manually curated MTI databases such as TarBase [69] or MirTarBase [70] contain only a limited set of possible interactions, available MTI prediction algorithms, e.g., miRanda for microrna.org [71,72], TargetScan [73], PITA [74] in the most cases consider only one or few important aspects of the complex biological setting of post-transcriptional regulation by miRNAs, like seed region complementarity [71,73], 3D structure and accessibility of binding site [74], thermodynamical stability of the miRNA–mRNA complex [71], evolutionary conservation of miRNA sequences [71], mutual evolution of miRNA seed regions and binding sites on targets [74], possible miRNA synergisms [75], or modulation of the MTIs by RNA-binding proteins [76]. These limitations imply a high degree of uncertainty when relying on predicted MTI data even in the case of MTI databases populated by machine learning algorithms like MirTarget for miRDB [77]. Thus to reduce the probability of false positive results or omitting crucial interactions, the miRNAtarget™ relies on multiple MTI databases and implements a simple yet powerful network theoretical approach as described previously [23].

### 3.4. Altered Cardiac Target mRNAs due to Sensory Neuropathy

We identified 15 target genes with high miRNA connectivity (≥degree 3) (Figure 1, dark blue spots). Out of these 15 predicted genes, we selected 4 target genes (*IGF-1*, *SLC2a-12*, *EIF-4e*, and *ULK-2*) based on the available literature related to myocardial function and/or diabetes for experimental validation. As target validation by qRT-PCR is considered to be a strong experimental evidence for miRNA–target interaction even by the most popular manually curated miRNA–target interaction database [78], we measured target expressions on the mRNA level by qRT-PCR. In case of all the 4 selected miRNA targets, we found significant upregulation at the mRNA level by qRT-PCR, which is in line with the expected expression changes, as all 4 targets were predicted to interact with at least two downregulated miRNAs. However, transfection-based experimental validation of the targets was out of the scope of our study.

Overexpression of *IGF-1* was found in the sensory neuropathic animals as a possible consequence of downregulation of *miR-1*, *miR-206*, and *miR-466b*. It has been described that IGF-1 affects the receptors of capsaicin (Transient Receptor Potential Vanilloid 1, TRPV1; [79,80]) and vice versa, capsaicin via TRPV1 receptors increases cardiac IGF-1 level [81]. Furthermore, IGF-1 levels have been shown to be reduced in diabetic peripheral neuropathy in the heart [82], extensively reviewed in [83]; however, we found here an upregulation of *IGF-1* gene in sensory neuropathy in the absence of hyperglycemia. Moreover, cardiac expression of *IGF-1* is altered in a variety of cardiovascular diseases [84,85], see [86] for a review. All of these pathologies are characterized by an initial upregulation of *IGF-1* and *IGF-1R*, which have been suggested to be involved in cardiac remodeling response [84,86]. Finally, a powerful regulatory link was recently shown in the heart between *IGF-1* and *miR-1* in a mouse model of pressure-overload-induced heart failure [87]. This *IGF-1*–*miR-1* connection was confirmed by our data in sensory neuropathy.

We found that *SLC2a-12* (or *GLUT-12*) was regulated by *miR-98*, *let-7a*, and *miR-466b*, and its myocardial mRNA level increased after systemic-capsaicin-treatment-induced sensory neuropathy. SLC2a-12 is an insulin sensitive glucose transporter [88,89]. Increased active SLC2a-12 content was observed at the surface of diabetic cardiomyocytes as a compensation of *GLUT-4* downregulation during diabetes [90]. Our results suggest that sensory neuropathy, as a complication of diabetes may lead to the downregulation of *let-7a* (see above) and thereby to overexpression of *SLC2a-12* in the heart.

In the present study, mRNA level of *EIF-4e* increased likely through the downregulation of *miR-1*, *miR-206*, and *miR-34b*. EIF-4e may affect protein synthesis as a downstream target of IGF-1–mTOR (mammalian target of rapamycin) pathway [91,92]. Dysregulation of this pathway may contribute to the development of both heart failure or type 2 diabetes, see [91] for a review.

In the present study, the expression of ULK-2, an autophagy activating protein, was increased as a possible consequence of downregulation of *miR-98*, *let-7a*, and *miR-34b*. It has already been shown in many cell types that capsaicin is able to induce autophagy [93,94]; however, the effect of high-dose capsaicin treatment on cardiac autophagy has not been investigated yet. Moreover, involvement of autophagy in cardiac complications of diabetes has also been described; however, its exact role is still not clear, see [95] for a review. In summary, our results suggest that autophagy may be altered in sensory neuropathy; however, further experiments are needed to clarify its exact mechanism.

### 3.5. Study Limitations

In the present study, the confounding effects of cardiovascular comorbidities and risk factors (e.g., age and sex) have not been studied, which is an obvious limitation of the study [96]; however, such experiments would have exceeded the scope of the present MS.

It is known that primary sensory afferents may influence blood glucose level due to several mechanisms, including morphological and functional interactions between the capsaicin receptor TRPV1 and the insulin receptor in the pancreas [97]. Moreover, we have found in the present study that *IGF-1* was overexpressed in sensory neuropathic animals; however, this finding was a result of an unbiased non-hypothesis driven workflow. Therefore, in the experimental protocol, we have not included glucose measurements; however, after revealing the results of the current unbiased study workflow, it is now evident that glucose measurements would have been an interesting addition to the present study.

In this study, we used only one method, i.e., qRT-PCR for the validation of the microRNA–mRNA target predictions, which is considered as a strong experimental evidence for microRNA–target interaction even according to the most popular manually curated microRNA–target interaction database [78]. Although several other methods including luciferase assays, target binding using variant seed sequence constructs, etc. are available, multiple target validation would have exceeded the scope of this study. Moreover, although, it is well-known that beyond mRNA degradation, miRNAs could also regulate protein expression via inhibition of translation by coupling to specific mRNA sequences [98], we have not investigated the effects of the altered miRNAs on translation or on protein level; therefore, our present study is restricted to translational downregulation of genes by miRNA targeting.

## 4. Materials and Methods

The present study conforms to the EU directive about the care and use of laboratory animals, published by the European Union (2010/63/EU) and was approved by the Ethics Committee for Animal Research of the University of Szeged. The animals were housed in individually ventilated cages (Sealsafe IVC system, Tecniplast S.p.a., Varese, Italy) which conform the size recommendations of the abovementioned EU guidelines. Litter material (Lignocell hygienic animal bedding) placed beneath the cage has been changed at least three times/week. The animal room was temperature controlled (22 ± 2 °C), had a 12-h light/dark cycle with lights on at 7 am to 7 pm. The animals were acclimatized in the housing facilities for 5 days prior to the start of the animal experiments. Animals were fed a standard rodent chow and filtered tap water ad libitum.

### 4.1. Induction of Selective Sensory Neuropathy by Systemic Capsaicin Treatment

It is well-established that selective sensory neuropathy can be induced by systemic capsaicin-induced desensitization. Systemic capsaicin treatment at appropriate doses induces a selective impairment of C-fiber primary sensory neurons expressing the capsaicin receptor TRPV1 [99,100,101]; which can be observed by, e.g., depletion of cardiac CGRP-containing afferent nerves [11]. Systemic capsaicin-desensitized animals have elevated chemical and heat pain thresholds, show no signs of pain, irritation or discomfort, and behave similar to controls [102].

Twelve male Wistar rats weighing 300–350 g were used throughout the experiments. General anesthesia was induced by the inhalation of 5% isoflurane and maintained with 2% for 10 min. Two minutes before the induction of volatile anesthesia, animals received 0.3 mg/kg nalbuphin to alleviate pain due to capsaicin treatment. Capsaicin (1% w/v, Fluka, Buchs, Switzerland) was administered subcutaneously (n = 6) on consecutive days at increasing doses of 10, 30, and 50 mg/kg, respectively, as described previously [11]. Ten minutes after capsaicin injection, isoflurane anesthesia was finished. Animals (n = 6) treated with the solvent for capsaicin (6% v/v ethanol and 8% v/v Tween 80 in physiological saline) served as controls. Seven days after the last injection of capsaicin, when depletion of TRPV1-expressing CGRP-containing myocardial sensory nerves is apparently complete [11], the body weights of the animals were measured. To confirm the development of sensory neuropathy in capsaicin-treated rats, eye-wipe behavioral test was performed before and after systemic capsaicin treatment [11,103,104]. Before capsaicin treatment, all the animals showed intensive eye wipe behavior after dropping 50 µL, 0.1% capsaicin into the left eye; however, 7 days after systemic capsaicin treatment, eye wipe behavior showed more than 10-fold decrease in capsaicin-treated animals.

Seven days after capsaicin treatment, hearts were excised under deep Na-pentobarbital (60 mg/kg ip.) anesthesia. To wash out blood, the hearts were perfused according to Langendorff for 5 min at 37 °C with Krebs–Henseleit bicarbonate buffer [105]. After perfusion, hearts were weighed and frozen immediately in liquid nitrogen, pulverized, and stored at −80 °C.

### 4.2. Transthoracic Echocardiography

The transthoracic echocardiography was performed in a blinded manner using a commercially available ultrasound machine (Vivid 7 Dimension, GE Medical Systems, Horten, Norway) equipped with a 2.5–3.5 MHz phased array sector scan probe, second harmonic technology, and coupled with tissue Doppler imaging (TDI). The left ventricular diameter, wall thickness, volume, and ejection fraction were measured from parasternal long-axis view using M-mode images. The left ventricular stroke volume was assessed from the diameter of the left ventricular outflow tract (LVOT), and time velocity integral of the LVOT was measured by pulsatile Doppler. LV diastolic function was determined from the velocities of mitral inflow by pulsed Doppler echocardiography complemented with mitral annular velocity of TDI.

### 4.3. Isolation of miRNAs

To 50 mg tissue powder, 190 μL proteinase K solution was added including 120 μL paraffin tissue lysis buffer (Roche, Mannheim, Germany). Samples were incubated at 55 °C for 30 min, and a two-step purification protocol was performed as described previously [106]. The quality and quantity of the isolated miRNA and total RNA were assessed spectrophotometrically (NanoDrop1000 Version 3.8.1. Thermo Fisher Scientific, Waltham, MA, USA) and with 2100 Bioanalyzer (Agilent Technologies Inc., Santa Clara, CA, USA). Three heart samples of the RNA extracted from the 6 different samples in each group were pooled, and the obtained 2 samples/group were assayed on the microarrays.

### 4.4. miRNA Microarray

Microarray analysis was conducted at Exiqon Services, Vedbaek, Denmark. The quality of the total RNA was verified by an Agilent 2100 Bioanalyzer profile (Agilent Technologies Inc., Santa Clara, CA, USA). Three-hundred and fifty nanograms of miRNA from both sample and control was labeled with Hy3™ and Hy5™ fluorescent label, respectively, using the miRCURY LNA™ microRNA Hi-Power Labeling Kit, Hy3™/Hy5™ (Exiqon, Vedbaek, Denmark) following the procedure described by the manufacturer. Briefly, Hy3™-labeled samples and a Hy5™-labeled control RNA sample were mixed pair-wise and hybridized to the miRCURY LNA™ microRNA Array 7th Gen (Exiqon, Vedbaek, Denmark), which contains capture probes targeting all microRNAs for human, mouse or rat, registered in the miRBASE 18.0. The hybridization was performed according to the miRCURY LNA™ microRNA Array Instruction manual using a Tecan HS4800™ hybridization station (Tecan Austria GmbH, Grödig, Austria). After hybridization, the microarray slides were scanned and stored in an ozone-free environment (Ozone level below 2.0 ppb) in order to prevent potential bleaching of the fluorescent dyes. The miRCURY LNA™ microRNA Array slides were scanned using the Agilent G2565BA Microarray Scanner System (Agilent Technologies Inc., Santa Clara, CA, USA), and the image analysis was carried out using the ImaGene^®^ 9 (miRCURY LNA™ microRNA Array Analysis Software, Exiqon, Vedbaek, Denmark). The quantified signals were background corrected (Normexp with offset value 10, see [107]), and normalized using the global Lowess (LOcally WEighted Scatterplot Smoothing) regression algorithm.

### 4.5. Validation of miRNA Array Results by qRT-PCR

To confirm microarray results, quantitative real-time PCR (qRT-PCR) miRNA Taqman assays were used (Thermo Fisher Scientific, Waltham, MA, USA). The reverse transcription reaction was performed with the TaqMan^®^ MicroRNA Reverse Transcription Kit (Applied Biosystems, Thermo Fisher Scientific, Waltham, MA, USA). Three-hundred and fifty nanograms from each sample was reverse transcribed in the presence of 5× RT TaqMan^®^ MicroRNA Assays (Applied Biosystems, Thermo Fisher Scientific, Waltham, MA, USA) as described previously [108]. Briefly, 8 μL reaction mixture contained 0.2 μL dNTPs, 1.5 μLMultiScribe ™ Reverse Transcriptase (50 U/μL), 0.8 μL 10× RT buffer, 0.9 μL MgCl_2_, 0.1 μL RNase inhibitor (20 U/μL), 1.5 μL 5× RT primer, and the template in a total volume of 3 μL. Reverse transcription was carried out with the following cycling parameters in a thermocycler (Bioneer Coorporation, Daejeon, Korea): 16 °C for 2 min, 42 °C for 1 min, 50 °C for 1 s, 45 cycles, then hold the samples on 85 °C for 5 min. After dilution with 64 μL of water, 9 μL of the diluted reaction mix was used as template in qRT-PCR. Reactions were performed on a RotorGene 3000 instrument (Corbett Research, Sydney, Australia) with the TaqMan protocol. Twenty microliters of reaction mixture contained 10 μL TaqMan^®^ Universal PCR Master Mix (Applied Biosystems, Thermo Fisher Scientific, Waltham, MA, USA), 1 μL of the TaqMan^®^ MicroRNA Assays, and 9 μL of the diluted cDNA. To further validate our data, we provided the relative expression level of each miRNA. We compared the average Ct values of the samples and subtracted the average Ct value of miR-1, one of the most abundant miRNA expressed in most tissues including the heart. miRNAs having values >10 correspond to low abundance miRNA, while <6 denotes for high abundant miRNA (for details, see Table S2).

### 4.6. Prediction of miRNA Targets

To identify target genes of the differentially expressed miRNAs identified by miRNA microarray, in silico target prediction approach was used to analyze miRNA–target interactions by miRNAtarget™ software (www.mirnatarget.com, Pharmahungary, Szeged, Hungary). In brief, the Norway rat specific version of publicly available predicted and experimentally validated databases, i.e., miRDB version 5.0 (released in August, 2014), microRNA.org (released in August, 2010) and miRTarBase 4.5 were queried by the miRNAtarget™, and score thresholds were applied to remove poor predictions (miRDB score ≤ 80.0 and mirSVR score ≥ −1.2) as described [23].

### 4.7. Selecting miRNA–Target Hubs

According to previous studies, the regulation of target genes by several miRNAs may be synergistic exerting a major effect on the bioavailability of target mRNAs [109]. To find such miRNA target hubs, a classical miRNA–target network was constructed by the miRNAtarget™ software in which the nodes represented miRNAs and target genes, while edges symbolized miRNA–target interactions. This miRNA–target network was visualized using the EntOptLayout plugin for Cytoscape [110].

### 4.8. Validation of Predicted miRNA Target Genes at mRNA Levels

Total RNA was isolated from control and sensory desensitized hearts with TRI reagent according to the manufacturer’s instructions (Molecular Research Center, Inc. Applied Biosystems, Thermo Fischer Scientific, Waltham, MA, USA), followed by reverse transcription (MMLV, Moloney Murine Leukemia Virus Reverse Transcriptase, 28025013, Thermo Fisher Scientific, Waltham, MA, USA). To detect the transcript levels of insulin-like growth factor-1 (*IGF-1*), solute carrier family 2 facilitated glucose transporter member 12 (*SLC2a-12*), eukaryotic translation initiation factor 4e (*EIF-4e*), and Unc-51 like autophagy activating kinase 2 (*ULK-2*), quantitative PCR was carried out with SYBR GREEN master mix (Fermentas, Thermo Fischer Scientific, Waltham, MA, USA) on a Light Cycler 1.5 (Roche, Applied Science, Penzberg, Upper Bavaria, Germany). As an internal control, *GAPDH* (Glycerinaldehyde-3-phosphate dehydrogenase) was amplified using the same heart samples. Primer pairs for *IGF-1*, *SLC2a-12*, *EIF-4e*, *ULK-2*, and *GAPDH* were designed against sequences of intron-spanning exons by Primer 3 Input software (Version 0.4.0; http://bioinfo.ut.ee/primer3-0.4.0/primer3/input.htm) and tested to avoid primer dimers, non-specific amplification, and self-priming by Primer-BLAST software (http://www.ncbi.nlm.nih.gov/tools/primer-blast/) (Table 6). Cycle conditions were set as an initial denaturation step for 10 min at 95 °C, followed by 45 cycles of 10 s at 95 °C for template denaturation, 10 s at 58 °C for annealing phase, and 10 s at 72 °C for extension. Specificity of the PCR products was confirmed by melting curve analysis followed by verification of the amplicon length on 1.5% agarose gels stained by ethidium bromide (Figure S2). To evaluate the results, crossing point values (Cp) were calculated. 2^−ΔΔCp^ methods were used to calculate relative mRNA levels of *IGF-1*, *ULK-2*, *SLC2a-12*, and *EIF-4e* normalized to *GAPDH* mRNA level.

### 4.9. Statistical Analysis

Six heart samples from each vehicle and capsaicin treated groups were analyzed by microRNA microarray. Three–three samples were pooled together and a total of 4 hybridization experiments were carried out to gain raw data for statistical analysis. Altogether 4 individual parallel gene activity comparisons were done to determine the average changes, standard deviations and *p*-values. Using two-tailed two-sample unequal variance Student’s t-test, the *p*-value was determined and used to find the significant gene expression changes. Gene expression ratio with *p*-value < 0.05 and log_2_ ratio < −0.6 or log_2_ ratio > 0.6 (~1.5 fold change) are considered as repression and overexpression, respectively. Changes in gene expression were plotted as log_2_ ratios of signal intensity values. Unpaired Student’s t-test was performed to compare morphometric and echocardiographic data as well as the data for qRT-PCR at mRNA level by using GraphPad Prism software (Version 6.04, Inc.; San Diego, CA, USA). All data showed normal distribution and are expressed as the mean ± SD. The level of *p* < 0.05 was considered significant.

## 5. Conclusions

This is the first demonstration that sensory neuropathy induced by capsaicin desensitization affects cardiac miRNA expression network targeting *IGF-1*, *SLC2a-12*, *EIF-4e*, and *ULK-2*, which may contribute to cardiac diastolic dysfunction induced by sensory neuropathy. These results further support the need for unbiased omics approach followed by in silico prediction and validation molecular targets to reveal novel pathomechanisms, as recently recommended by a position paper from the European Society for Cardiology [22].

## Figures and Tables

**Table 1:** Effect of sensory neuropathy on body and heart weights.

	BW 0	BW 10	ΔBW (10–0)	HW	HW/BW
	(g)	(g)	(g)	(mg)	(‰)
**Control**	339 ± 25	380 ± 40	41 ± 20	1167 ± 116	3.1 ± 0.2
**Sensory Neuropathy**	328 ± 16	335 ± 17 *	7 ± 5 **	972 ± 98 *	2.9 ± 0.2

BW 0, body weight at baseline; BW 10, body weight at 7th day after last capsaicin injection; and HW, heart weight. Values are shown as means ± SD (n = 6 in each group; * *p* < 0.05 versus control, ** *p* < 0.01 versus control; unpaired Student’s t-test).

**Table 2:** Effect of sensory neuropathy on myocardial morphology and function assessed by transthoracic echocardiography.

Parameter	Control	Sensory Neuropathy	*p*-Value
Heart rate	412 ± 32	441 ± 35	0.30
EDD (cm)	0.69 ± 0.05	0.62 ± 0.03	0.03 *
ESD (cm)	0.40 ± 0.10	0.36 ± 0.05	0.46
IVS (cm)	0.15 ± 0.01	0.13 ± 0.01	0.04 *
PW (cm)	0.16 ± 0.01	0.15 ± 0.01	0.12
EDV (ml)	0.70 ± 0.18	0.54 ± 0.07	0.14
ESV (ml)	0.18 ± 0.10	0.12 ± 0.05	0.30
SV (EDV–ESV)	0.54 ± 0.07	0.42 ± 0.07	0.03 *
LVEF (%)	75.83 ± 9.89	77.57 ± 8.46	0.79
LVFS (%)	41.14 ± 9.77	41.89 ± 7.35	0.90
MVVTI (cm)	4.14 ± 0.36	3.23 ± 0.75	0.04 *
E (cm/sec)	80.67 ± 14.40	65.2 ± 11.39	0.11
A (cm/sec)	59.50 ± 27.87	53.2 ± 31.22	0.76
Ea (cm/sec)	6.33 ± 1.25	6.20 ± 3.19	0.93
Aa (cm/sec)	5.50 ± 1.71	6.40 ± 1.36	0.41
Sa (cm/sec)	5.00 ± 1.15	5.60 ± 1.85	0.57
E/Ea	13.07 ± 3.02	13.03 ± 5.44	0.99
Ea/Aa	1.26 ± 0.44	0.98 ± 0.48	0.23

Data are shown as mean ± SD., n = 5–6; * *p* < 0.05; unpaired Student’s t-test. EDD, end-diastolic diameter; ESD, end-systolic diameter; IVS, interventricular septum; PW, posterior wall thickness; EDV, end-diastolic volume; ESV, end-systolic volume; SV, stroke volume; EF, ejection fraction; FS, fractional shortening; MVVTI, mitral valve velocity time index; E, wave for early diastolic filling; A, wave for atrial filling.

**Table 3:** Expression of selected microRNAs (miRNAs) by microarray analysis.

miRNA	Average log2 Change Sensory Neuropathy/Control	Log2 SD	Regulation
*rno-miR-344b-1-3p*	−1.95 *	0.47	down
*rno-miR-466b-1-3p*	−1.10 *	0.49	down
*rno-miR-98-5p*	−1.07	1.26	down
*rno-let-7a-5p*	−1.03	1.21	down
*rno-miR-1-3p*	−0.88	1.19	down
*rno-miR-206-3p*	−0.86	1.23	down
*rno-miR-34b-3p*	0.63	0.46	up
*rno-miR-181a-2-3p*	0.75 *	0.27	up

Gene expression ratio with *p*-value < 0.05 and log2 ratio < −0.6 or log2 ratio > 0.6 (~1.5 fold change) are considered as repression and overexpression, respectively. SD, standard deviation; * *p* < 0.05 versus control; unpaired unequal variance Student’s t-test.

**Table 4:** Validation of microarray analysis by qRT-PCR.

miRNA	Average log2 Change Sensory Neuropathy/Control	log2 SD	Fold Change	Regulation	Confirmation
***rno-miR-344b-1-3p***	n.d.	n.d.	n.d.	n.d.	n.d.
***rno-miR-466b-1-3p***	−3.19 *	1.59	−9.13 *	down	yes
***rno-miR-98-5p***	−2.45 **	1.19	−5.48 **	down	yes
***rno-let-7a-5p***	−2.03 **	0.54	−4.07 **	down	yes
***rno-miR-1-3p***	−2.85 **	1.03	−7.21 **	down	yes
***rno-miR-206-3p***	−5.19 **	1.58	−36.42 **	down	yes
***rno-miR-34b-3p***	−2.93 **	1.76	−7.60 **	down	no
***rno-miR-181a-2-3p***	3.81 **	1.55	14.03 **	up	yes

SD, standard deviation; n.d., non-detectable; * *p* < 0.05, ** *p* < 0.01 versus control; two tailed two sample unequal variance Student’s t-test.

**Table 5:** Selected target genes indicating their miRNA connections by plus sign (+).

Target		Regulated by					
Abbreviation	Name	*miR-466b*	*miR-98*	*let-7a*	*miR-1*	*miR-206*	*miR-34b*
*IGF-1*	Insulin-like growth factor-1	+			+	+	
*SLC2a-12*	Solute carrier family 2 facilitated glucose transporter member 12	+	+	+			
*EIF-4e*	Eukaryotic translation initiation factor 4e				+	+	+
*ULK-2*	Unc-51 like autophagy activating kinase 2		+	+			+

All miRNAs for the selected gene targets showed downregulation in the sensory neuropathy group as compared to the control. Plus signs (+) show the related miRNAs for each target mRNA molecules.

**Table 6:** Primer properties used in qRT-PCR for determination of transcript levels.

Target	Accession Number	Forward Primer	Reverse Primer	Efficiency	Product Size (bp)
*IGF-1*	M15481.1	CAGTTCGTGTGTGGACCAAG	GAGTCTTGGGCATGTCAGTG	1.966	211
*SLC2a-12*	NM_001107451.1	CCGAGACAAAGGGATGCTCT	CCTTGTAAGTCCTGCCACCA	1.704	251
*EIF-4e*	NM_053974.2	CAAGCAAACCTTCGGTTGAT	CTCCCCGTTTGTTTTTCTCA	1.743	163
*ULK-2*	NM_001191645.1	CACAGAACGACCAATGGATG	TTGTTCGAAGGACCATGTGA	2.00	161
*GAPDH*	NM_017008.4	GCATCTTCTTGTGCAGTGCC	GAGAAGGCAGCCCTGGTAAC	1.991	105

*IGF-1*, insulin-like growth factor 1; *SLC2a-12*, solute carrier family 2 facilitated glucose transporter member 12; *EIF-4e*, eukaryotic translation initiation factor 4e; *ULK-2*, Unc-51 like autophagy activating kinase 2; *GAPDH*, glyceraldehyde-3-phosphate dehydrogenase; and bp, base pair.

## Supplementary Materials

Supplementary materials can be found at https://www.mdpi.com/1422-0067/20/4/991/s1.

## Abbreviations

**AMI**	acute myocardial infarction
**EIF-4e**	eukaryotic translation initiation factor 4e
**eNOS**	endothelial nitrogen monoxide synthase
**GAPDH**	glycerinaldehyde-3-phosphate dehydrogenase
**GLUT-4**	glucose transporter type 4
**HF**	heart failure
**IGF-1**	insulin-like growth factor-1
**miRNA**	MicroRNA
**MMLV**	Moloney Murine Leukemia Virus Reverse Transcriptase
**mRNA**	messenger RNAs
**MTI**	microRNA-target interaction
**mTOR**	mammalian target of rapamycin
**NO**	nitrogen monoxide
**qRT-PCR**	quantitative real-time PCR
**SLC2a-12 (GLUT-12)**	solute carrier family 2 facilitated glucose transporter member 12
**SOD1**	superoxide dismutase-1
**TRPV1**	transient receptor potential vanilloid 1
**ULK-2**	Unc-51 like autophagy activating kinase 2
