# Supplementary material for: Sensory Neuropathy Affects Cardiac miRNA Expression Network Targeting IGF-1, SLC2a-12, EIF-4e, and ULK-2 mRNAs
Source: Int J Mol Sci. 2019 Feb 25;20(4):991. doi: 10.3390/ijms20040991 (PMC6412859; doi:10.3390/ijms20040991)
Supplement: Supplementary file 1 [file ijms-20-00991-s001.pdf]

# Sensory neuropathy affects cardiac miRNA expression network targeting *IGF-1*, *SLC2a-12*, *EIF-4e*, and *ULK-2* mRNAs

**Table S1.** Full data set for the microRNA (miRNA)–target gene network analysis.

| Abbreviatio | Entrez ID | Gene Target Full Name of Targets | Access. Number | Associated miRNAs 1 | 2 | 3 | 4 |
|---|---|---|---|---|---|---|---|
| LOC100125362 | 100125362 | hypothetical protein LOC100125362 | NM_001103354 | rno-miR-344b-1-3p | | | |
| Kdm6a | 100310845 | lysine demethylase 6A | XM_002727527 | rno-miR-466b-1 | | | |
| Tmem167a | 100359823 | transmembrane protein 167A | XM_002728992 | rno-let-7a | rno-miR-98 | | |
| Tyk2 | 100361294 | tyrosine kinase 2 | NM_001257347 | rno-let-7a | rno-miR-98 | | |
| Kmt2d | 100362634 | lysine methyltransferase 2D | XM_006257392 | rno-let-7a | rno-miR-98 | | |
| Cdkl5 | 100362725 | cyclin-dependent kinase-like 5 | XM_006256909 | rno-miR-1 | rno-miR-206 | | |
| Ddx55 | 100362764 | DEAD-box helicase 55 | NM_001271326 | rno-miR-466b-1 | | | |
| LOC100362819 | 100362819 | autism susceptibility candidate 2-like | XM_003752582 | rno-miR-466b-1 | | | |
| LOC100910224 | 100910224 | olfactory receptor 8D1-like | XM_003750491 | rno-miR-344b-1-3p | | | |
| LOC100910506 | 100910506 | peripheral plasma membrane protein CASK-like | XM_006256651 | rno-miR-466b-1 | | | |
| LOC100910807 | 100910807 | transcriptional regulator Kaiso-like | XM_003752135 | rno-miR-181a-2 | | | |
| LOC100910990 | 100910990 | copine-1-like | XM_006235453 | rno-miR-466b-1 | | | |
| Med7 | 100911235 | mediator complex subunit 7 | NM_001286183 | rno-miR-34b | | | |
| LOC100911428 | 100911428 | cyclic AMP-dependent transcription factor ATF-3-like | XM_006250492 | rno-miR-1 | rno-miR-206 | | |
| LOC100911548 | 100911548 | SPRY domain-containing SOCS box protein 2-like | XM_006244626 | rno-miR-1 | | | |
| LOC100912483 | 100912483 | uncharacterized LOC100912483 | XM_006246854 | rno-miR-1 | rno-miR-206 | | |
| LOC102548535 | 102548535 | uncharacterized LOC102548535 | XM_006242247 | rno-miR-181a-2 | | | |
| LOC102552077 | 102552077 | uncharacterized LOC102552077 | XM_006224696 | rno-let-7a | | | |
| LOC102555920 | 102555920 | sperm motility kinase X-like | XM_006235205 | rno-let-7a | | | |
| LOC102556920 | 102556920 | uncharacterized LOC102556920 | XM_006225294 | rno-let-7a | rno-miR-98 | | |
| Nrg1 | 112400 | neuregulin 1 | NM_001271118 | rno-miR-466b-1 | | | |
| Ndst2 | 114002 | N-deacetylase/N-sulfotransferase 2 | NM_001105740 | rno-let-7a | rno-miR-98 | | |
| Hcn2 | 114244 | hyperpolarization activated cyclic nucleotide-gated potassium channel 2 | NM_053684 | rno-miR-1 | | | |
| Dnajc14 | 114481 | DnaJ heat shock protein family (Hsp40) member C14 | NM_053690 | rno-miR-466b-1 | | | |
| Dag1 | 114489 | dystroglycan 1 | NM_053697 | rno-miR-466b-1 | | | |
| Pax3 | 114502 | paired box 3 | NM_053710 | rno-miR-1 | rno-miR-206 | | |
| Clasp2 | 114514 | cytoplasmic linker associated protein 2 | NM_053722 | rno-let-7a | rno-miR-98 | | |
| Strn3 | 114520 | striatin 3 | NM_001029897 | rno-miR-34b | | | |
| Wif1 | 114557 | Wnt inhibitory factor 1 | NM_053738 | rno-miR-466b-1 | | | |
| Snap29 | 116500 | synaptosomal-associated protein 29 | NM_053810 | rno-miR-466b-1 | | | |
| E2f5 | 116651 | E2F transcription factor 5 | XM_006224083 | rno-let-7a | rno-miR-1 | rno-miR-206 | rno-miR-98 |
| Rere | 116665 | arginine-glutamic acid dipeptide (RE) repeats | NM_053885 | rno-miR-344b-1-3p | | | |
| Apbb3 | 117026 | amyloid beta precursor protein binding family B member 3 | NM_053957 | rno-let-7a | rno-miR-98 | | |
| Eif4e | 117045 | eukaryotic translation initiation factor 4E | NM_053974 | rno-miR-1 | rno-miR-206 | rno-miR-34b | |
| Hivep1 | 117140 | human immunodeficiency virus type I enhancer binding protein 1 | NM_001105751 | rno-miR-344b-1-3p | rno-miR-466b-1 | | |
| Tra2b | 117259 | transformer 2 beta homolog (Drosophila) | NM_057119 | rno-miR-1 | rno-miR-181a-2 | rno-miR-206 | |
| Hnrnpu | 117280 | heterogeneous nuclear ribonucleoprotein U | NM_057139 | rno-miR-1 | rno-miR-206 | | |
| Hnrnpk | 117282 | heterogeneous nuclear ribonucleoprotein K | NM_057141 | rno-miR-181a-2 | | | |
| Kif1b | 117548 | kinesin family member 1B | NM_057200 | rno-miR-466b-1 | | | |
| Tpm3 | 117557 | tropomyosin 3 | NM_173111 | rno-miR-1 | rno-miR-206 | | |

| | | | | | | |
|---|---|---|---|---|---|---|
| Flt3 | 140635 | fms-related tyrosine kinase 3 | NM_001100822 | rno-miR-34b | | |
| Rplp1 | 140661 | ribosomal protein, large, P1 | NM_001007604 | rno-miR-1 | | |
| Daxx | 140926 | death-domain associated protein | NM_080891 | rno-miR-181a-2 | | |
| Ikbkap | 140934 | inhibitor of kappa light polypeptide gene enhancer in B-cells, kinase complex-associated protein | NM_080899 | rno-let-7a | rno-miR-98 | |
| Tmprss2 | 156435 | transmembrane protease, serine 2 | NM_130424 | rno-let-7a | rno-miR-98 | |
| Prkcd | 170538 | protein kinase C, delta | NM_133307 | rno-miR-466b-1 | | |
| Olr59 | 170816 | olfactory receptor 59 | NM_173293 | rno-miR-344b-1-3p | | |
| Map4k3 | 170920 | mitogen-activated protein kinase kinase kinase kinase 3 | NM_133407 | rno-let-7a | rno-miR-98 | |
| Itga2 | 170921 | integrin alpha 2 | XM_345156 | rno-miR-344b-1-3p | | |
| Shank2 | 171093 | SH3 and multiple ankyrin repeat domains 2 | NM_133440 | rno-miR-344b-1-3p | | |
| Slc31a1 | 171135 | solute carrier family 31 member 1 | NM_133600 | rno-miR-181a-2 | | |
| Ehhadh | 171142 | enoyl-CoA, hydratase/3-hydroxyacyl CoA dehydrogenase | NM_133606 | rno-let-7a | rno-miR-98 | |
| Slc2a13 | 171147 | solute carrier family 2 member 13 | NM_133611 | rno-miR-34b | | |
| Taf9b | 171152 | TAF9B RNA polymerase II, TATA box binding protein (TBP)-associated factor | NM_133615 | rno-let-7a | rno-miR-98 | |
| Ap1g1 | 171494 | adaptor-related protein complex 1, gamma 1 subunit | NM_134460 | rno-miR-466b-1 | | |
| Hip1 | 192154 | huntingtin interacting protein 1 | NM_001100475 | rno-let-7a | rno-miR-98 | |
| Dnajc21 | 192210 | DnaJ heat shock protein family (Hsp40) member C21 | NM_138856 | rno-miR-34b | | |
| Ppp1r2 | 192361 | protein phosphatase 1, regulatory (inhibitor) subunit 2 | NM_138823 | rno-miR-344b-1-3p | rno-miR-466b-1 | |
| Adrb2 | 24176 | adrenoceptor beta 2 | NM_012492 | rno-let-7a | rno-miR-98 | |
| Bcl2 | 24224 | B-cell CLL/lymphoma 2 | NM_016993 | rno-miR-1 | | |
| Bdnf | 24225 | brain-derived neurotrophic factor | NM_001270635 | rno-miR-1 | rno-miR-206 | |
| Calm1 | 24242 | calmodulin 1 | NM_031969 | rno-miR-1 | | |
| Calm3 | 24244 | calmodulin 3 | NM_012518 | rno-miR-1 | | |
| Edn1 | 24323 | endothelin 1 | NM_012548 | rno-miR-1 | rno-miR-206 | |
| Ets1 | 24356 | ETS proto-oncogene 1, transcription factor | NM_012555 | rno-miR-1 | rno-miR-206 | |
| G6pd | 24377 | glucose-6-phosphate dehydrogenase | NM_017006 | rno-miR-1 | rno-miR-206 | |
| Gja1 | 24392 | gap junction protein, alpha 1 | NM_012567 | rno-miR-1 | rno-miR-206 | |
| Grm1 | 24414 | glutamate receptor, metabotropic 1 | NM_017011 | rno-miR-466b-1 | | |
| Hmox1 | 24451 | heme oxygenase 1 | NM_012580 | rno-let-7a | | |
| Igf1 | 24482 | insulin-like growth factor 1 | NM_178866 | rno-miR-1 | rno-miR-206 | rno-miR-466b-1 |
| Jun | 24516 | jun proto-oncogene | NM_021835 | rno-miR-466b-1 | | |
| Acvr1c | 245921 | activin A receptor type 1C | NM_139090 | rno-let-7a | rno-miR-98 | |
| Nupl1 | 245922 | nucleoporin 58 | NM_139091 | rno-miR-466b-1 | | |
| Nras | 24605 | neuroblastoma RAS viral (v-ras) oncogene homolog | NM_080766 | rno-let-7a | rno-miR-98 | |
| Scd1 | 246074 | stearoyl-CoA desaturase | NM_139192 | rno-let-7a | rno-miR-98 | |
| Ptpn4 | 246116 | protein tyrosine phosphatase, non-receptor type 4 | NM_001100479 | rno-miR-1 | rno-miR-206 | |
| Bmf | 246142 | Bcl2 modifying factor | NM_139258 | rno-miR-466b-1 | | |
| Wdr44 | 246152 | WD repeat domain 44 | NM_001100823 | rno-miR-344b-1-3p | | |
| Pla2g4a | 24653 | phospholipase A2 group IVA | NM_133551 | rno-miR-1 | rno-miR-206 | |
| Prkar2b | 24679 | protein kinase cAMP-dependent type 2 regulatory subunit beta | NM_001030020 | rno-miR-34b | | |
| Vamp2 | 24803 | vesicle-associated membrane protein 2 | NM_012663 | rno-miR-1 | rno-miR-206 | |
| Syt2 | 24805 | synaptotagmin 2 | NM_012665 | rno-miR-181a-2 | | |
| Stx1b | 24923 | syntaxin 1B | NM_012700 | rno-miR-466b-1 | | |
| Adm | 25026 | adrenomedullin | NM_012715 | rno-miR-344b-1-3p | | |

| | | | | | | | |
|---|---|---|---|---|---|---|---|
| Tagln | 25123 | transgelin | NM_031549 | rno-let-7a | rno-miR-98 | | |
| Ncl | 25135 | nucleolin | NM_012749 | rno-miR-1 | rno-miR-206 | | |
| Map1a | 25152 | microtubule-associated protein 1A | NM_030995 | rno-miR-1 | rno-miR-206 | | |
| Itpr1 | 25262 | inositol 1,4,5-trisphosphate receptor, type 1 | NM_001270597 | rno-miR-466b-1 | | | |
| Emp1 | 25314 | epithelial membrane protein 1 | NM_012843 | rno-miR-181a-2 | | | |
| Adarb1 | 25367 | adenosine deaminase, RNA-specific, B1 | NM_001111055 | rno-miR-466b-1 | | | |
| Faslg | 25385 | Fas ligand | NM_012908 | rno-let-7a | rno-miR-98 | | |
| Sparcl1 | 25434 | SPARC like 1 | NM_012946 | rno-miR-1 | rno-miR-206 | | |
| Myo1e | 25484 | myosin IE | NM_173101 | rno-miR-1 | rno-miR-206 | | |
| Ywhaq | 25577 | tyrosine 3-monooxygenase/tryptophan 5-monooxygenase activation protein, theta | NM_013053 | rno-miR-1 | rno-miR-206 | | |
| Ptprz1 | 25613 | protein tyrosine phosphatase, receptor type Z1 | NM_013080 | rno-miR-1 | rno-miR-206 | | |
| Crem | 25620 | cAMP responsive element modulator | NM_001271246 | rno-miR-466b-1 | | | |
| Adrb3 | 25645 | adrenoceptor beta 3 | NM_013108 | rno-let-7a | rno-miR-98 | | |
| Fn1 | 25661 | fibronectin 1 | NM_019143 | rno-miR-1 | rno-miR-206 | | |
| Nr3c2 | 25672 | nuclear receptor subfamily 3, group C, member 2 | NM_013131 | rno-miR-466b-1 | | | |
| Anxa5 | 25673 | annexin A5 | NM_013132 | rno-miR-1 | | | |
| Snx27 | 260323 | sorting nexin family member 27 | NM_001110151 | rno-miR-466b-1 | | | |
| Cops2 | 261736 | COP9 signalosome subunit 2 | NM_153297 | rno-miR-1 | rno-miR-206 | | |
| Hspa4 | 266759 | heat shock protein family A member 4 | NM_153629 | rno-miR-1 | | | |
| Phax | 286917 | phosphorylated adaptor for RNA export | NM_173133 | rno-miR-1 | rno-miR-206 | | |
| Unc13c | 286931 | unc-13 homolog C (C. elegans) | NM_173146 | rno-miR-466b-1 | | | |
| Olr1271 | 286959 | olfactory receptor 1271 | NM_173300 | rno-miR-344b-1-3p | | | |
| Cramp1l | 287127 | cramped chromatin regulator homolog 1 | XM_006246049 | rno-miR-466b-1 | | | |
| Zbtb4 | 287441 | zinc finger and BTB domain containing 4 | XM_006246843 | rno-miR-206 | | | |
| Ddx5 | 287765 | DEAD-box helicase 5 | NM_001007613 | rno-miR-1 | rno-miR-206 | | |
| Hic2 | 287940 | hypermethylated in cancer 2 | NM_001105862 | rno-let-7a | rno-miR-98 | | |
| Naa50 | 288108 | N(alpha)-acetyltransferase 50, NatE catalytic subunit | NM_001105881 | rno-miR-466b-1 | | | |
| Wrb | 288233 | tryptophan rich basic protein | NM_199373 | rno-miR-466b-1 | | | |
| Mis18a | 288272 | MIS18 kinetochore protein A | NM_001127523 | rno-miR-466b-1 | | | |
| Alkbh4 | 288587 | alkB homolog 4, lysine demethylase | NM_001105920 | rno-let-7a | rno-miR-98 | | |
| Srsf9 | 288701 | serine/arginine-rich splicing factor 9 | NM_001009255 | rno-miR-1 | rno-miR-206 | | |
| Hmcn1 | 289094 | hemicentin 1 | NM_001271292 | rno-miR-1 | rno-miR-206 | | |
| Lrrc52 | 289199 | leucine rich repeat containing 52 | NM_001077434 | rno-miR-181a-2 | | | |
| Fras1 | 289486 | Fraser extracellular matrix complex subunit 1 | NM_001191595 | rno-let-7a | rno-miR-98 | | |
| Gnpda2 | 289608 | glucosamine-6-phosphate deaminase 2 | NM_001106005 | rno-miR-1 | rno-miR-206 | | |
| RGD1563065 | 289633 | NACHT and WD repeat domain containing 2 | XM_223426 | rno-miR-466b-1 | | | |
| Pla2g3 | 289733 | phospholipase A2, group III | NM_001106015 | rno-let-7a | rno-miR-98 | | |
| Psmc6 | 289990 | proteasome 26S subunit, ATPase 6 | NM_001100509 | rno-miR-466b-1 | | | |
| Cldn10 | 290485 | claudin 10 | NM_001106058 | rno-miR-344b-1-3p | | | |
| Dctn6 | 290798 | dynactin subunit 6 | NM_001106085 | rno-miR-344b-1-3p | | | |
| Mcur1 | 291034 | mitochondrial calcium uniporter regulator 1 | XM_214448 | rno-miR-466b-1 | | | |
| Snn | 29140 | stannin | NM_001034083 | rno-let-7a | rno-miR-98 | | |
| Snx2 | 291464 | sorting nexin 2 | NM_001106135 | rno-miR-1 | rno-miR-206 | | |
| Matr3 | 29150 | matrin 3 | NM_019149 | rno-miR-1 | rno-miR-206 | | |
| Psmg2 | 291539 | proteasome (prosome, macropain) assembly chaperone 2 | NM_001106138 | rno-miR-344b-1-3p | | | |
| Rit2 | 291713 | Ras-like without CAAX 2 | NM_001013060 | rno-miR-1 | rno-miR-206 | | |

| | | | | | | | |
|---|---|---|---|---|---|---|---|
| Orc6 | 291927 | origin recognition complex, subunit 6 | NM_001033690 | rno-miR-344b-1-3p | | | |
| Pgrmc1 | 291948 | progesterone receptor membrane component 1 | NM_021766 | rno-let-7a | rno-miR-98 | | |
| Gch1 | 29244 | GTP cyclohydrolase 1 | NM_024356 | rno-miR-1 | rno-miR-206 | | |
| Psg29 | 292666 | pregnancy-specific glycoprotein 29 | NM_001025641 | rno-miR-1 | rno-miR-206 | | |
| Meox2 | 29279 | mesenchyme homeobox 2 | NM_017149 | rno-miR-1 | rno-miR-206 | | |
| Chsy1 | 292999 | chondroitin sulfate synthase 1 | NM_001106268 | rno-miR-1 | rno-miR-206 | | |
| Cpeb1 | 293056 | cytoplasmic polyadenylation element binding protein 1 | NM_001106276 | rno-miR-1 | rno-miR-206 | | |
| Uqcrc2 | 293448 | ubiquinol cytochrome c reductase core protein 2 | NM_001006970 | rno-miR-466b-1 | | | |
| Kctd13 | 293497 | potassium channel tetramerization domain containing 13 | NM_198736 | rno-miR-1 | rno-miR-206 | | |
| Zfp275 | 293849 | zinc finger protein 275 | NM_001106343 | rno-let-7a | rno-miR-98 | | |
| March5 | 294079 | membrane associated ring-CH-type finger 5 | NM_001106372 | rno-miR-466b-1 | | | |
| Rasgrp1 | 29434 | RAS guanyl releasing protein 1 | NM_019211 | rno-let-7a | rno-miR-98 | | |
| Ranbp2 | 294429 | RAN binding protein 2 | NM_001191604 | rno-let-7a | rno-miR-98 | | |
| Fam26e | 294431 | family with sequence similarity 26, member E | NM_001024977 | rno-miR-1 | rno-miR-206 | | |
| Pald1 | 294508 | phosphatase domain containing, paladin 1 | NM_001034128 | rno-let-7a | rno-miR-98 | | |
| Ttc33 | 294774 | tetratricopeptide repeat domain 33 | NM_001106414 | rno-miR-344b-1-3p | | | |
| March6 | 294862 | membrane associated ring-CH-type finger 6 | XM_001065952 | rno-miR-34b | | | |
| Ift80 | 295106 | intraflagellar transport 80 | NM_001013911 | rno-miR-1 | rno-miR-206 | | |
| Lppr4 | 295401 | phospholipid phosphatase related 4 | NM_001001508 | rno-miR-1 | rno-miR-206 | | |
| Neurog2 | 295475 | neurogenin 2 | XM_001076231 | rno-miR-466b-1 | | | |
| Rif1 | 295602 | replication timing regulatory factor 1 | XM_003753723 | rno-miR-466b-1 | | | |
| Upp2 | 295620 | uridine phosphorylase 2 | NM_001106481 | rno-miR-466b-1 | | | |
| Fign | 295649 | fidgetin | NM_001106484 | rno-miR-181a-2 | | | |
| Tgfbr1 | 29591 | transforming growth factor, beta receptor 1 | NM_012775 | rno-let-7a | rno-miR-98 | | |
| Secisbp2l | 296115 | SECIS binding protein 2-like | NM_001168527 | rno-miR-466b-1 | | | |
| Snph | 296267 | syntaphilin | NM_001106525 | rno-miR-181a-2 | | | |
| Svs3b | 296354 | seminal vesicle secretory protein 3B | NM_001102417 | rno-miR-466b-1 | | | |
| Cask | 29647 | calcium/calmodulin-dependent serine protein kinase (MAGUK family) | NM_022184 | rno-miR-466b-1 | | | |
| Helz2 | 296474 | helicase with zinc finger 2, transcriptional coactivator | XM_230961 | rno-miR-1 | rno-miR-206 | | |
| Adam10 | 29650 | ADAM metallopeptidase domain 10 | NM_019254 | rno-miR-344b-1-3p | | | |
| Fam73b | 296623 | family with sequence similarity 73, member B | NM_001106566 | rno-miR-1 | rno-miR-206 | | |
| Phf19 | 296653 | PHD finger protein 19 | NM_001106570 | rno-miR-344b-1-3p | | | |
| Psma3 | 29670 | proteasome subunit alpha 3 | NM_017280 | rno-miR-1 | rno-miR-206 | | |
| Srpk2 | 296753 | SRSF protein kinase 2 | NM_001106575 | rno-miR-466b-1 | | | |
| Aass | 296925 | aminoadipate-semialdehyde synthase | NM_001100963 | rno-miR-466b-1 | | | |
| Zfp282 | 297065 | zinc finger protein 282 | NM_001106592 | rno-let-7a | rno-miR-98 | | |
| Kcnj2 | 29712 | potassium voltage-gated channel subfamily J member 2 | NM_017296 | rno-miR-1 | | | |
| Slc8a1 | 29715 | solute carrier family 8 member A1 | NM_001270772 | rno-miR-1 | | | |
| Ccdc174 | 297458 | coiled-coil domain containing 174 | NM_001009659 | rno-miR-181a-2 | | | |
| Setd5 | 297514 | SET domain containing 5 | NM_001106614 | rno-miR-466b-1 | | | |
| Dsel | 297865 | dermatan sulfate epimerase-like | XM_006221430 | rno-miR-466b-1 | | | |
| Zdhhc21 | 298184 | zinc finger, DHHC-type containing 21 | NM_001039009 | rno-miR-34b | rno-miR-466b-1 | | |
| Smap2 | 298500 | small ArfGAP2 | NM_001100669 | rno-let-7a | rno-miR-98 | | |
| Clspn | 298534 | claspin | NM_001106687 | rno-miR-466b-1 | | | |
| Tmem50a | 298552 | transmembrane protein 50A | NM_001127525 | rno-miR-181a-2 | | | |
| Six4 | 299138 | SIX homeobox 4 | NM_001106739 | rno-miR-466b-1 | | | |

| | | | | | | | |
|---|---|---|---|---|---|---|---|
| Ylpm1 | 299199 | YLP motif containing 1 | NM_001271258 | rno-miR-466b-1 | | | |
| Cpsf6 | 299811 | cleavage and polyadenylation specific factor 6 | NM_001106785 | rno-miR-344b-1-3p | | | |
| Lrig3 | 299830 | leucine-rich repeats and immunoglobulin-like domains 3 | XM_216905 | rno-let-7a | rno-miR-98 | | |
| Gtse1 | 300126 | G-2 and S-phase expressed 1 | NM_001130500 | rno-miR-466b-1 | | | |
| Gxylt1 | 300173 | glucoside xylosyltransferase 1 | NM_001100887 | rno-let-7a | rno-miR-466b-1 | rno-miR-98 | |
| Ccdc82 | 300359 | coiled-coil domain containing 82 | NM_001007660 | rno-miR-466b-1 | | | |
| | | | | | | | |
| Adamts15 | 300474 | ADAM metallopeptidase with thrombospondin type 1 motif, 15 | NM_001106810 | rno-let-7a | rno-miR-98 | | |
| Arcn1 | 300674 | archain 1 | NM_001007662 | rno-miR-1 | rno-miR-206 | | |
| Arih1 | 300756 | ariadne RBR E3 ubiquitin protein ligase 1 | NM_001013108 | rno-let-7a | rno-miR-98 | | |
| Ptplad1 | 300783 | 3-hydroxyacyl-CoA dehydratase 3 | NM_001106831 | rno-miR-1 | rno-miR-206 | | |
| Tmem108 | 300967 | transmembrane protein 108 | XM_006243685 | rno-miR-344b-1-3p | | | |
| Wdr6 | 301007 | WD repeat domain 6 | NM_001006988 | rno-miR-1 | rno-miR-206 | | |
| Trim42 | 301106 | tripartite motif-containing 42 | NM_001013955 | rno-miR-466b-1 | | | |
| Acer1 | 301118 | alkaline ceramidase 1 | NM_001106875 | rno-let-7a | rno-miR-98 | | |
| Tfap2b | 301285 | transcription factor AP-2 beta | NM_001106896 | rno-let-7a | | | |
| RGD1310553 | 301374 | similar to expressed sequence AI597479 | NM_001008517 | rno-miR-466b-1 | | | |
| Mbnl3 | 302492 | muscleblind-like splicing regulator 3 | NM_001106949 | rno-miR-181a-2 | | | |
| Unkl | 302987 | unkempt family like zinc finger | XM_006246058 | rno-miR-34b | | | |
| Crebrf | 303016 | CREB3 regulatory factor | NM_001277157 | rno-miR-181a-2 | rno-miR-466b-1 | | |
| Trim41 | 303088 | tripartite motif-containing 41 | NM_001134737 | rno-let-7a | rno-miR-98 | | |
| Ulk2 | 303206 | unc-51 like autophagy activating kinase 2 | NM_001191645 | rno-let-7a | rno-miR-34b | rno-miR-98 | |
| Rab11fip4 | 303337 | RAB11 family interacting protein 4 (class II) | NM_001107023 | rno-let-7a | rno-miR-98 | | |
| Mmd | 303439 | monocyte to macrophage differentiation-associated | NM_001007673 | rno-miR-466b-1 | | | |
| Kat7 | 303470 | lysine acetyltransferase 7 | NM_181081 | rno-miR-181a-2 | | | |
| Osbpl7 | 303497 | oxysterol binding protein-like 7 | NM_001107044 | rno-miR-1 | rno-miR-206 | | |
| Smurf2 | 303614 | SMAD specific E3 ubiquitin protein ligase 2 | NM_001107061 | rno-miR-466b-1 | | | |
| Klhl24 | 303803 | kelch-like family member 24 | NM_181473 | rno-miR-466b-1 | | | |
| Igf2bp2 | 303824 | insulin-like growth factor 2 mRNA binding protein 2 | NM_001270598 | rno-let-7a | rno-miR-98 | | |
| Bcl6 | 303836 | B-cell CLL/lymphoma 6 | NM_001107084 | rno-miR-466b-1 | | | |
| Abhd10 | 303953 | abhydrolase domain containing 10 | NM_001123352 | rno-miR-466b-1 | | | |
| Arl13b | 304037 | ADP-ribosylation factor like GTPase 13B | NM_001107101 | rno-miR-344b-1-3p | | | |
| Son | 304092 | Son DNA binding protein | NM_001170328 | rno-miR-466b-1 | | | |
| Cldn8 | 304124 | claudin 8 | NM_001037774 | rno-miR-466b-1 | | | |
| Rwdd2b | 304132 | RWD domain containing 2B | NM_001100559 | rno-miR-181a-2 | | | |
| Nrip1 | 304157 | nuclear receptor interacting protein 1 | NM_001100560 | rno-miR-466b-1 | | | |
| Tnpo2 | 304670 | transportin 2 | NM_001107166 | rno-miR-1 | rno-miR-206 | | |
| Ccnt2 | 304758 | cyclin T2 | NM_001107171 | rno-miR-466b-1 | | | |
| Pbx1 | 304947 | pre-B-cell leukemia homeobox 1 | NM_001134862 | rno-let-7a | rno-miR-98 | | |
| Lin54 | 305171 | lin-54 DREAM MuvB core complex component | NM_001100564 | rno-miR-466b-1 | | | |
| Mtmr3 | 305482 | myotubularin related protein 3 | NM_001012038 | rno-miR-466b-1 | | | |
| Papolg | 305586 | poly(A) polymerase gamma | NM_001107244 | rno-miR-466b-1 | | | |
| Abhd6 | 305795 | abhydrolase domain containing 6 | NM_001007680 | rno-miR-1 | | | |
| RGD1306353 | 305911 | zinc finger MYM-type containing 5 | XM_006221946 | rno-miR-466b-1 | | | |
| Fndc3a | 306022 | fibronectin type III domain containing 3a | NM_001107278 | rno-let-7a | rno-miR-1 | rno-miR-206 | rno-miR-98 |
| Duxbl1 | 306226 | double homeobox B-like 1 | XM_006252679 | rno-miR-466b-1 | | | |
| Pde12 | 306231 | phosphodiesterase 12 | NM_001013998 | rno-let-7a | rno-miR-98 | | |

| | | | | | | | |
|---|---|---|---|---|---|---|---|
| Pbrm1 | 306254 | polybromo 1 | XM_006252690 | rno-miR-344b-1-3p | rno-miR-466b-1 | | |
| Ercc6 | 306274 | excision repair cross-complementation group 6 | NM_001107296 | rno-let-7a | rno-miR-98 | | |
| Psd3 | 306380 | pleckstrin and Sec7 domain containing 3 | XM_006253047 | rno-let-7a | rno-miR-344b-1-3p | rno-miR-466b-1 | rno-miR-98 |
| Kat6a | 306571 | lysine acetyltransferase 6A | NM_001100570 | rno-miR-344b-1-3p | rno-miR-466b-1 | | |
| Papd7 | 306672 | PAP associated domain containing 7 | NM_001107333 | rno-miR-344b-1-3p | | | |
| Ripk1 | 306886 | receptor interacting serine/threonine kinase 1 | NM_001107350 | rno-miR-466b-1 | | | |
| Cdk13 | 306998 | cyclin-dependent kinase 13 | NM_001271296 | rno-miR-344b-1-3p | | | |
| Cndp1 | 307212 | carnosine dipeptidase 1 (metallopeptidase M20 family) | NM_001007687 | rno-miR-1 | rno-miR-206 | | |
| Zfp608 | 307296 | zinc finger protein 608 | NM_001107378 | rno-miR-181a-2 | | | |
| Cep120 | 307302 | centrosomal protein 120 | NM_001191697 | rno-let-7a | rno-miR-98 | | |
| Fam210a | 307343 | family with sequence similarity 210, member A | NM_001007688 | rno-miR-466b-1 | | | |
| Ammecr1l | 307526 | AMMECR1 like | NM_001107399 | rno-miR-1 | rno-miR-206 | | |
| Gan | 307893 | gigaxonin | NM_001107434 | rno-miR-181a-2 | | | |
| Map10 | 307948 | microtubule-associated protein 10 | NM_001277391 | rno-miR-466b-1 | | | |
| Slc2a12 | 308028 | solute carrier family 2 member 12 | NM_001107451 | rno-let-7a | rno-miR-466b-1 | rno-miR-98 | |
| Map3k4 | 308106 | mitogen activated protein kinase kinase kinase 4 | NM_001107456 | rno-miR-466b-1 | | | |
| Lats1 | 308265 | large tumor suppressor kinase 1 | NM_001134543 | rno-miR-466b-1 | | | |
| Adamtsl3 | 308787 | ADAMTS-like 3 | NM_001107533 | rno-miR-1 | rno-miR-206 | | |
| RGD1305254 | 308797 | cell migration inducing protein, hyaluronan binding | XM_006229510 | rno-miR-466b-1 | | | |
| Prkrir | 308845 | THAP domain containing 12 | NM_001191630 | rno-miR-1 | rno-miR-206 | | |
| Fchsd2 | 308864 | FCH and double SH3 domains 2 | NM_001107539 | rno-miR-344b-1-3p | | | |
| Dock1 | 309081 | dedicator of cyto-kinesis 1 | NM_001143858 | rno-miR-181a-2 | | | |
| Clrn3 | 309082 | clarin 3 | NM_001014026 | rno-let-7a | rno-miR-98 | | |
| Slc25a22 | 309111 | solute carrier family 25 member 22 | NM_001014027 | rno-miR-1 | rno-miR-206 | | |
| RGD1310016 | 309306 | RIC1 homolog, RAB6A GEF complex partner 1 | XM_006231255 | rno-miR-466b-1 | | | |
| Uhrf2 | 309331 | ubiquitin like with PHD and ring finger domains 2 | NM_001107585 | rno-let-7a | rno-miR-98 | | |
| Tmem2 | 309400 | transmembrane protein 2 | NM_001107596 | rno-let-7a | rno-miR-98 | | |
| Cpeb3 | 309510 | cytoplasmic polyadenylation element binding protein 3 | XM_006231338 | rno-miR-344b-1-3p | | | |
| Sec63 | 309858 | SEC63 homolog, protein translocation regulator | NM_001107637 | rno-miR-1 | rno-miR-206 | | |
| Tmem161b | 309953 | transmembrane protein 161B | XM_226652 | rno-miR-466b-1 | | | |
| Mef2a | 309957 | myocyte enhancer factor 2a | NM_001014035 | rno-miR-1 | | | |
| Gcnt4 | 310011 | glucosaminyl (N-acetyl) transferase 4, core 2 | XM_006223982 | rno-let-7a | rno-miR-98 | | |
| Rictor | 310131 | RPTOR independent companion of MTOR, complex 2 | XM_226812 | rno-let-7a | rno-miR-98 | | |
| Zc2hc1a | 310244 | zinc finger, C2HC-type containing 1A | NM_001107661 | rno-miR-466b-1 | | | |
| Eif5a2 | 310261 | eukaryotic translation initiation factor 5A2 | NM_001100697 | rno-miR-344b-1-3p | | | |
| Jade1 | 310352 | jade family PHD finger 1 | NM_001107670 | rno-miR-466b-1 | | | |
| Nhlrc3 | 310416 | NHL repeat containing 3 | XM_227139 | rno-let-7a | rno-miR-98 | | |
| Rapgef2 | 310533 | Rap guanine nucleotide exchange factor 2 | NM_001107684 | rno-miR-181a-2 | | | |
| Golph3l | 310669 | golgi phosphoprotein 3-like | NM_001007698 | rno-miR-1 | rno-miR-206 | | |
| Rsbn1 | 310749 | round spermatid basic protein 1 | NM_001191710 | rno-miR-466b-1 | | | |
| Lrig2 | 310753 | leucine-rich repeats and immunoglobulin-like domains 2 | NM_001107710 | rno-let-7a | rno-miR-98 | | |
| Cttnbp2nl | 310760 | CTTNBP2 N-terminal like | NM_001107712 | rno-miR-1 | rno-miR-206 | | |
| Slc25a24 | 310791 | solute carrier family 25 member 24 | NM_001127544 | rno-let-7a | rno-miR-98 | | |
| Usp33 | 310960 | ubiquitin specific peptidase 33 | NM_001191094 | rno-miR-1 | rno-miR-206 | rno-miR-344b-1-3p | |
| Ctnnd1 | 311163 | catenin delta 1 | NM_001107740 | rno-miR-181a-2 | | | |
| Rrbp1 | 311483 | ribosome binding protein 1 | XM_006235130 | rno-miR-1 | rno-miR-206 | | |
| Zfp512b | 311721 | zinc finger protein 512B | NM_001107809 | rno-let-7a | rno-miR-98 | | |

| | | | | | | | |
|---|---|---|---|---|---|---|---|
| Pbx3 | 311876 | pre-B-cell leukemia homeobox 3 | NM_001107834 | rno-let-7a | rno-miR-98 | | |
| Scaf11 | 312030 | SR-related CTD-associated factor 11 | NM_001271170 | rno-miR-1 | rno-miR-206 | | |
| Kbtbd2 | 312372 | kelch repeat and BTB domain containing 2 | NM_001107861 | rno-miR-466b-1 | | | |
| Smarcad1 | 312398 | SWI/SNF-related, matrix-associated actin-dependent regulator of chromatin, subfamily a, containing DEAD/H box 1 | NM_001107864 | rno-let-7a | rno-miR-98 | | |
| Ubap2 | 313169 | ubiquitin-associated protein 2 | NM_001107928 | rno-miR-466b-1 | | | |
| Ptprd | 313278 | protein tyrosine phosphatase, receptor type, D | XM_006225366 | rno-miR-466b-1 | | | |
| Stag2 | 313304 | stromal antigen 2 | NM_001173507 | rno-miR-466b-1 | | | |
| Kank4 | 313385 | KN motif and ankyrin repeat domains 4 | NM_001107947 | rno-miR-1 | rno-miR-206 | | |
| Mier1 | 313418 | mesoderm induction early response 1, transcriptional regulator | NM_001131012 | rno-let-7a | rno-miR-98 | | |
| Pdik1l | 313609 | PDLIM1 interacting kinase 1 like | NM_001107984 | rno-miR-1 | rno-miR-206 | | |
| Fbxl12 | 313782 | F-box and leucine-rich repeat protein 12 | NM_001025700 | rno-let-7a | rno-miR-98 | | |
| Birc6 | 313876 | baculoviral IAP repeat-containing 6 | NM_001170596 | rno-miR-466b-1 | | | |
| Ncoa1 | 313929 | nuclear receptor coactivator 1 | NM_001108012 | rno-miR-466b-1 | | | |
| Cmpk2 | 314004 | cytidine/uridine monophosphate kinase 2 | NM_001108017 | rno-miR-1 | rno-miR-206 | | |
| Cbll1 | 314028 | Cbl proto-oncogene-like 1, E3 ubiquitin protein ligase | NM_001108018 | rno-miR-466b-1 | | | |
| Fbxo33 | 314157 | F-box protein 33 | NM_001108023 | rno-miR-344b-1-3p | | | |
| Daam1 | 314212 | dishevelled associated activator of morphogenesis 1 | NM_001108030 | rno-miR-466b-1 | | | |
| Six6 | 314221 | SIX homeobox 6 | NM_001108032 | rno-miR-466b-1 | | | |
| Zfyve26 | 314265 | zinc finger FYVE-type containing 26 | NM_001108038 | rno-let-7a | rno-miR-98 | | |
| Gpr132 | 314480 | G protein-coupled receptor 132 | NM_001170595 | rno-let-7a | rno-miR-98 | | |
| Ap3d1 | 314633 | adaptor-related protein complex 3, delta 1 subunit | NM_001100719 | rno-miR-1 | rno-miR-206 | | |
| Cdk17 | 314743 | cyclin-dependent kinase 17 | NM_001108082 | rno-miR-344b-1-3p | | | |
| Eea1 | 314764 | early endosome antigen 1 | NM_001108086 | rno-let-7a | rno-miR-98 | | |
| Frs2 | 314850 | fibroblast growth factor receptor substrate 2 | NM_001108097 | rno-miR-1 | rno-miR-206 | | |
| Mon2 | 314894 | MON2 homolog, regulator of endosome-to-Golgi trafficking | NM_001040176 | rno-miR-1 | rno-miR-206 | | |
| Phf20l1 | 314964 | PHD finger protein 20-like 1 | NM_001271439 | rno-miR-466b-1 | | | |
| Fbxo43 | 315034 | F-box protein 43 | NM_001012117 | rno-miR-344b-1-3p | | | |
| Josd1 | 315134 | Josephin domain containing 1 | NM_001025009 | rno-miR-1 | rno-miR-206 | | |
| Twf1 | 315265 | twinfilin actin-binding protein 1 | NM_001008521 | rno-miR-1 | rno-miR-206 | | |
| Nxt2 | 315352 | nuclear transport factor 2-like export factor 2 | NM_001108120 | rno-miR-1 | rno-miR-181a-2 | rno-miR-206 | rno-miR-466b-1 |
| Jrkl | 315417 | jerky-like | NM_001108122 | rno-miR-344b-1-3p | | | |
| Arhgap32 | 315530 | Rho GTPase activating protein 32 | XM_008766074 | rno-miR-1 | | | |
| Npat | 315666 | nuclear protein, co-activator of histone transcription | NM_001108147 | rno-miR-466b-1 | | | |
| Peak1 | 315686 | pseudopodium-enriched atypical kinase 1 | NM_001108149 | rno-miR-466b-1 | | | |
| RGD1305464 | 315702 | similar to human chromosome 15 open reading frame 39 | NM_001025011 | rno-let-7a | rno-miR-98 | | |
| Adpgk | 315722 | ADP-dependent glucokinase | NM_001100723 | rno-miR-1 | rno-miR-206 | | |
| Kif23 | 315740 | kinesin family member 23 | NM_001108155 | rno-miR-466b-1 | | | |
| Cgnl1 | 315795 | cingulin-like 1 | NM_001108164 | rno-let-7a | rno-miR-98 | | |
| Zfp280d | 315798 | zinc finger protein 280D | NM_001108165 | rno-miR-1 | rno-miR-206 | | |
| Rfx7 | 315804 | regulatory factor X, 7 | NM_001127490 | rno-miR-466b-1 | | | |
| Klhl31 | 315833 | kelch-like family member 31 | NM_001108170 | rno-let-7a | rno-miR-98 | | |
| Msl2 | 315959 | male-specific lethal 2 homolog (Drosophila) | XM_006226541 | rno-miR-466b-1 | | | |
| Arih2 | 316005 | ariadne RBR E3 ubiquitin protein ligase 2 | NM_001012275 | rno-miR-181a-2 | | | |

| | | | | | | | |
|---|---|---|---|---|---|---|---|
| Rev1 | 316344 | REV1, DNA directed polymerase | NM_001108213 | rno-miR-466b-1 | | | |
| B3gnt7 | 316583 | UDP-GlcNAc:betaGal beta-1,3-N-acetylglucosaminyltransferase 7 | NM_001012134 | rno-let-7a | rno-miR-98 | | |
| Apool | 317191 | apolipoprotein O-like | NM_001014105 | rno-miR-466b-1 | | | |
| Mageb18 | 317270 | MAGE family member B18 | NM_001044246 | rno-miR-344b-1-3p | | | |
| Stk40 | 360230 | serine/threonine kinase 40 | NM_183056 | rno-let-7a | rno-miR-98 | | |
| Ublcp1 | 360514 | ubiquitin-like domain containing CTD phosphatase 1 | NM_001014117 | rno-miR-181a-2 | | | |
| Rnft1 | 360595 | ring finger protein, transmembrane 1 | XM_006247119 | rno-miR-344b-1-3p | | | |
| Msi2 | 360596 | musashi RNA-binding protein 2 | XM_006220777 | rno-miR-344b-1-3p | | | |
| Ccdc43 | 360637 | coiled-coil domain containing 43 | NM_001100728 | rno-miR-181a-2 | | | |
| Limd2 | 360646 | LIM domain containing 2 | NM_001025715 | rno-let-7a | rno-miR-98 | | |
| Mob1b | 360920 | MOB kinase activator 1B | NM_001108357 | rno-miR-344b-1-3p | rno-miR-34b | | |
| Smek2 | 360993 | protein phosphatase 4, regulatory subunit 3B | NM_001108367 | rno-miR-1 | rno-miR-206 | | |
| Ktn1 | 361029 | kinectin 1 | XM_006221908 | rno-miR-1 | rno-miR-206 | | |
| RGD1308117 | 361066 | similar to 9930012K11Rik protein | NM_001134571 | rno-let-7a | rno-miR-98 | | |
| Ndfip2 | 361089 | Nedd4 family interacting protein 2 | NM_001108390 | rno-miR-344b-1-3p | | | |
| Tmem110 | 361110 | transmembrane protein 110 | NM_198774 | rno-miR-344b-1-3p | | | |
| Cbfb | 361391 | core-binding factor, beta subunit | NM_001013191 | rno-miR-344b-1-3p | rno-miR-466b-1 | | |
| Tppp | 361466 | tubulin polymerization promoting protein | NM_001108461 | rno-miR-1 | rno-miR-206 | | |
| Ube3a | 361585 | ubiquitin protein ligase E3A | NM_001191837 | rno-miR-466b-1 | | | |
| Fam168a | 361614 | family with sequence similarity 168, member A | NM_001108494 | rno-miR-466b-1 | | | |
| Eif4g2 | 361628 | eukaryotic translation initiation factor 4, gamma 2 | NM_001017374 | rno-let-7a | rno-miR-98 | | |
| Patl1 | 361736 | protein associated with topoisomerase II homolog 1 (yeast) | NM_001108520 | rno-miR-181a-2 | | | |
| Fam160b1 | 361774 | family with sequence similarity 160, member B1 | XM_001064355 | rno-miR-466b-1 | | | |
| Hace1 | 361866 | HECT domain and ankyrin repeat containing, E3 ubiquitin protein ligase 1 | NM_001108539 | rno-miR-1 | rno-miR-206 | | |
| Trim2 | 361970 | tripartite motif-containing 2 | NM_001108552 | rno-miR-1 | | | |
| Lmo4 | 362051 | LIM domain only 4 | NM_001009708 | rno-miR-466b-1 | rno-miR-98 | | |
| Nr6a1 | 362125 | nuclear receptor subfamily 6, group A, member 1 | XM_006234131 | rno-let-7a | rno-miR-98 | | |
| Hnrnpa3 | 362152 | heterogeneous nuclear ribonucleoprotein A3 | NM_001111294 | rno-miR-1 | rno-miR-206 | | |
| Atg13 | 362164 | autophagy related 13 | NM_001271212 | rno-miR-1 | rno-miR-206 | | |
| Api5 | 362170 | apoptosis inhibitor 5 | NM_001127379 | rno-miR-466b-1 | | | |
| Gpcpd1 | 362219 | glycerophosphocholine phosphodiesterase 1 | NM_198779 | rno-miR-466b-1 | | | |
| Pard6b | 362279 | par-6 family cell polarity regulator beta | NM_001108609 | rno-let-7a | rno-miR-98 | | |
| Cdk14 | 362316 | cyclin-dependent kinase 14 | NM_001108617 | rno-miR-1 | rno-miR-206 | | |
| Mxd1 | 362391 | max dimerization protein 1 | NM_001100749 | rno-miR-466b-1 | | | |
| Rpusd3 | 362416 | RNA pseudouridylate synthase domain containing 3 | NM_001108641 | rno-let-7a | rno-miR-98 | | |
| Tmtc1 | 362465 | transmembrane and tetratricopeptide repeat containing 1 | XM_001075561 | rno-miR-181a-2 | | | |
| Runx1t1 | 362489 | RUNX1 translocation partner 1 | NM_001108657 | rno-miR-34b | rno-miR-466b-1 | | |
| Zfp462 | 362522 | zinc finger protein 462 | XM_342840 | rno-miR-466b-1 | | | |
| Trappc3 | 362599 | trafficking protein particle complex 3 | NM_001008376 | rno-miR-1 | rno-miR-206 | | |
| Gpatch3 | 362615 | G patch domain containing 3 | XM_342933 | rno-let-7a | rno-miR-98 | | |
| Cebpz | 362686 | CCAAT/enhancer binding protein zeta | NM_001108701 | rno-miR-1 | rno-miR-206 | | |
| Tmem178a | 362691 | transmembrane protein 178A | NM_001004282 | rno-miR-1 | rno-miR-206 | | |
| Yipf4 | 362699 | Yip1 domain family, member 4 | NM_001009712 | rno-miR-1 | rno-miR-206 | | |
| Wdr43 | 362703 | WD repeat domain 43 | NM_001037791 | rno-miR-466b-1 | | | |

| | | | | | | |
|---|---|---|---|---|---|---|
| Seli | 362713 | selenoprotein I | NM_001134754 | rno-miR-181a-2 | | |
| Pax9 | 362741 | paired box 9 | NM_001039539 | rno-miR-181a-2 | | |
| Tmx1 | 362751 | thioredoxin-related transmembrane protein 1 | NM_001024800 | rno-miR-1 | rno-miR-206 | |
| Cwf19l2 | 362804 | CWF19-like 2, cell cycle control (S. pombe) | NM_001135003 | rno-miR-466b-1 | | |
| Hsp90b1 | 362862 | heat shock protein 90, beta, member 1 | NM_001012197 | rno-miR-1 | rno-miR-206 | |
| Tarbp2 | 363006 | TAR (HIV-1) RNA binding protein 2 | NM_001034941 | rno-let-7a | rno-miR-98 | |
| Uhrf1bp1l | 363009 | UHRF1 binding protein 1-like | NM_001108753 | rno-miR-1 | rno-miR-206 | |
| Zbtb44 | 363035 | zinc finger and BTB domain containing 44 | NM_001034942 | rno-miR-466b-1 | | |
| Nphp3 | 363126 | nephronophthisis 3 (adolescent) | NM_001191882 | rno-let-7a | rno-miR-98 | |
| Rassf1 | 363140 | Ras association domain family member 1 | NM_001007754 | rno-miR-466b-1 | | |
| Ccdc12 | 363151 | coiled-coil domain containing 12 | NM_001108783 | rno-miR-466b-1 | | |
| Wdr48 | 363164 | WD repeat domain 48 | NM_001135895 | rno-miR-1 | rno-miR-206 | rno-miR-34b |
| Tcaim | 363169 | T cell activation inhibitor, mitochondrial | NM_001110838 | rno-miR-34b | | |
| Bzw1 | 363232 | basic leucine zipper and W2 domains 1 | NM_198789 | rno-let-7a | rno-miR-98 | |
| Atg16l1 | 363278 | autophagy related 16-like 1 | NM_001108809 | rno-miR-344b-1-3p | | |
| Abi3bp | 363767 | ABI family member 3 binding protein | XM_006248231 | rno-miR-466b-1 | | |
| Yod1 | 363982 | YOD1 deubiquitinase | BC107904 | rno-let-7a | rno-miR-98 | |
| Smim14 | 364154 | small integral membrane protein 14 | NM_001037792 | rno-miR-1 | rno-miR-206 | |
| Tmem55b | 364298 | transmembrane protein 55B | NM_001014233 | rno-miR-1 | rno-miR-206 | |
| Ranbp9 | 364686 | RAN binding protein 9 | XM_002725261 | rno-miR-34b | | |
| Nkd1 | 364952 | naked cuticle homolog 1 (Drosophila) | NM_001271381 | rno-let-7a | rno-miR-98 | |
| Lcor | 365462 | ligand dependent nuclear receptor corepressor | XM_006223759 | rno-miR-344b-1-3p | | |
| Zfr | 365703 | zinc finger RNA binding protein | NM_001270972 | rno-miR-466b-1 | | |
| Cers6 | 366065 | ceramide synthase 6 | XM_006234308 | rno-miR-466b-1 | | |
| Slc5a9 | 366441 | solute carrier family 5 member 9 | NM_001108974 | rno-let-7a | rno-miR-98 | |
| Sh2d5 | 366489 | SH2 domain containing 5 | XM_001070433 | rno-miR-181a-2 | | |
| Tbc1d15 | 366896 | TBC1 domain family, member 15 | XM_006241353 | rno-miR-1 | rno-miR-206 | |
| Arid2 | 366980 | AT-rich interaction domain 2 | XM_006226280 | rno-miR-344b-1-3p | | |
| Mgat4a | 367252 | mannosyl (alpha-1,3-)-glycoprotein beta-1,4-N-acetylglucosaminyltransferase, isozyme A | NM_001012225 | rno-miR-1 | rno-miR-206 | |
| Ankib1 | 368062 | ankyrin repeat and IBR domain containing 1 | NM_001134781 | rno-miR-1 | rno-miR-206 | |
| aicda | 399679 | activation-induced cytidine deaminase | NM_001100779 | rno-miR-466b-1 | | |
| Rnf5 | 407784 | ring finger protein 5, E3 ubiquitin protein ligase | NM_001109025 | rno-let-7a | rno-miR-98 | |
| Pdcd10 | 494345 | programmed cell death 10 | NM_001009542 | rno-miR-1 | rno-miR-181a-2 | rno-miR-206 |
| LOC497899 | 497899 | similar to hypothetical protein 4930503F14 | NM_001017472 | rno-let-7a | rno-miR-98 | |
| RGD1559732 | 497912 | butyrophilin like 10 | XM_003750838 | rno-miR-466b-1 | | |
| Blzf1 | 498266 | basic leucine zipper nuclear factor 1 | NM_001017494 | rno-miR-344b-1-3p | | |
| Lcorl | 498385 | ligand dependent nuclear receptor corepressor-like | XM_001058603 | rno-miR-344b-1-3p | | |
| Psme4 | 498433 | proteasome activator subunit 4 | NM_001025140 | rno-miR-181a-2 | | |
| Akap11 | 498549 | A-kinase anchoring protein 11 | NM_012773 | rno-miR-1 | rno-miR-206 | |
| Nrg3 | 498596 | neuregulin 3 | XM_006252793 | rno-miR-181a-2 | | |
| RGD1562037 | 498764 | similar to OTTHUMP00000046255 | XM_006222413 | rno-miR-466b-1 | | |
| Ankrd29 | 498823 | ankyrin repeat domain 29 | NM_001190372 | rno-miR-1 | rno-miR-206 | |
| Ahrr | 498999 | aryl-hydrocarbon receptor repressor | NM_001024285 | rno-miR-466b-1 | | |
| Zfp583 | 499068 | zinc finger protein 583 | NM_001134609 | rno-let-7a | rno-miR-98 | |
| Acer3 | 499210 | alkaline ceramidase 3 | XM_001065019 | rno-let-7a | rno-miR-98 | |
| Nmrk1 | 499330 | nicotinamide riboside kinase 1 | NM_001024292 | rno-miR-466b-1 | | |

| | | | | | | | |
|---|---|---|---|---|---|---|---|
| Fam178a | 499360 | SMC5-SMC6 complex localization factor 2 | NM_001134612 | rno-let-7a | rno-miR-98 | | |
| Ccdc169 | 499618 | coiled-coil domain containing 169 | NM_001109184 | rno-miR-466b-1 | | | |
| Prpf38b | 499691 | pre-mRNA processing factor 38B | NM_001024305 | rno-let-7a | rno-miR-98 | | |
| Cldn12 | 500000 | claudin 12 | NM_001100813 | rno-let-7a | rno-miR-98 | | |
| Foxp2 | 500037 | forkhead box P2 | NM_001271104 | rno-miR-181a-2 | rno-miR-466b-1 | | |
| Creb5 | 500131 | cAMP responsive element binding protein 5 | NM_001134621 | rno-miR-344b-1-3p | | | |
| Mmrn1 | 500152 | multimerin 1 | XM_006224950 | rno-miR-344b-1-3p | | | |
| Styk1 | 500340 | serine/threonine/tyrosine kinase 1 | XM_006237464 | rno-let-7a | rno-miR-98 | | |
| Ybx1 | 500538 | Y box binding protein 1 | NM_031563 | rno-let-7a | | | |
| Lin28a | 500562 | lin-28 homolog A | NM_001109269 | rno-let-7a | rno-miR-98 | | |
| Tmem198b | 500762 | transmembrane protein 198b | NM_001109281 | rno-let-7a | rno-miR-98 | | |
| Fam19a5 | 500915 | family with sequence similarity 19 (chemokine (C-C motif)-like), member A5 | NM_001191991 | rno-miR-344b-1-3p | | | |
| Mdfi | 501097 | MyoD family inhibitor | NM_001109301 | rno-let-7a | rno-miR-98 | | |
| Bex4 | 501624 | brain expressed, X-linked 4 | NM_001037554 | rno-miR-1 | rno-miR-206 | | |
| RGD1566084 | 501795 | proline rich 36 | XM_006248788 | rno-miR-1 | rno-miR-206 | | |
| Coro1c | 501841 | coronin 1C | NM_001109327 | rno-miR-1 | rno-miR-206 | | |
| Lrch1 | 502020 | leucine-rich repeats and calponin homology (CH) domain containing 1 | NM_001134727 | rno-miR-1 | rno-miR-206 | | |
| Slc35f1 | 502421 | solute carrier family 35, member F1 | NM_001109338 | rno-miR-1 | rno-miR-206 | | |
| Srsf11 | 502603 | serine/arginine-rich splicing factor 11 | NM_001035255 | rno-miR-344b-1-3p | | | |
| Serf2 | 502663 | small EDRK-rich factor 2 | NM_001098782 | rno-miR-466b-1 | | | |
| Rerg | 502916 | RAS-like, estrogen-regulated, growth-inhibitor | XM_006225116 | rno-miR-466b-1 | | | |
| Sowahc | 503306 | sosondowah ankyrin repeat domain family member C | NM_001109364 | rno-miR-1 | rno-miR-206 | | |
| Calm2 | 50663 | calmodulin 2 | NM_017326 | rno-miR-1 | | | |
| Cltc | 54241 | clathrin heavy chain | NM_019299 | rno-miR-466b-1 | | | |
| Gata4 | 54254 | GATA binding protein 4 | NM_144730 | rno-miR-1 | | | |
| Cnn3 | 54321 | calponin 3 | NM_019359 | rno-miR-1 | rno-miR-206 | | |
| Apln | 58812 | apelin | NM_031612 | rno-miR-466b-1 | | | |
| Nckap1 | 58823 | NCK-associated protein 1 | NM_031618 | rno-miR-34b | | | |
| Mapk6 | 58840 | mitogen-activated protein kinase 6 | NM_031622 | rno-let-7a | rno-miR-98 | | |
| Klf6 | 58954 | Kruppel-like factor 6 | NM_031642 | rno-miR-466b-1 | | | |
| Azin1 | 58961 | antizyme inhibitor 1 | NM_022585 | rno-miR-1 | rno-miR-206 | | |
| Nme6 | 58964 | NME/NM23 nucleoside diphosphate kinase 6 | NM_001191884 | rno-let-7a | rno-miR-98 | | |
| Grk6 | 59076 | G protein-coupled receptor kinase 6 | NM_001112712 | rno-miR-1 | rno-miR-206 | | |
| Hcn4 | 59266 | hyperpolarization activated cyclic nucleotide-gated potassium channel 4 | NM_021658 | rno-miR-1 | | | |
| Ip6k2 | 59268 | inositol hexakisphosphate kinase 2 | NM_021660 | rno-miR-1 | rno-miR-206 | | |
| Trdn | 59299 | triadin | NM_021666 | rno-miR-344b-1-3p | | | |
| Clock | 60447 | clock circadian regulator | NM_021856 | rno-miR-1 | rno-miR-206 | rno-miR-34b | |
| Exoc5 | 60627 | exocyst complex component 5 | NM_022204 | rno-miR-344b-1-3p | | | |
| Ncoa4 | 619385 | nuclear receptor coactivator 4 | NM_001034007 | rno-miR-344b-1-3p | | | |
| Fam104a | 619573 | family with sequence similarity 104, member A | NM_001034958 | rno-miR-466b-1 | | | |
| Hspd1 | 63868 | heat shock protein family D member 1 | NM_022229 | rno-miR-1 | rno-miR-206 | | |
| Cd244 | 64025 | CD244 molecule | NM_022259 | rno-let-7a | rno-miR-98 | | |
| Casp7 | 64026 | caspase 7 | NM_022260 | rno-miR-1 | | | |
| Ccnd2 | 64033 | cyclin D2 | NM_022267 | rno-miR-98 | | | |

| | | | | | | |
|---|---|---|---|---|---|---|
| Snx16 | 64088 | sorting nexin 16 | NM_022289 | rno-let-7a | rno-miR-98 | |
| Pi4ka | 64161 | phosphatidylinositol 4-kinase alpha | NM_022301 | rno-miR-1 | rno-miR-206 | |
| Ppib | 64367 | peptidylprolyl isomerase B | NM_022536 | rno-miR-1 | rno-miR-206 | |
| Ndrg4 | 64457 | NDRG family member 4 | NM_001271092 | rno-miR-466b-1 | | |
| Bpnt1 | 64473 | 3'(2'), 5'-bisphosphate nucleotidase 1 | NM_171990 | rno-miR-344b-1-3p | | |
| Tspan2 | 64521 | tetraspanin 2 | NM_022589 | rno-miR-466b-1 | | |
| Cnbp | 64530 | CCHC-type zinc finger, nucleic acid binding protein | NM_022598 | rno-miR-1 | rno-miR-206 | |
| Snap23 | 64630 | synaptosomal-associated protein 23 | NM_022689 | rno-miR-34b | | |
| Csnk1g3 | 64823 | casein kinase 1, gamma 3 | NM_022855 | rno-miR-466b-1 | | |
| Crisp1 | 64827 | cysteine-rich secretory protein 1 | NM_022859 | rno-let-7a | | |
| Synpr | 66030 | synaptoporin | NM_023974 | rno-miR-466b-1 | | |
| | | | | | | |
| Hmg1l1 | 679571 | high-mobility group (nonhistone chromosomal) protein 1-like 1 | NM_001109373 | rno-miR-344b-1-3p | | |
| Slc17a4 | 679784 | solute carrier family 17, member 4 | NM_001271214 | rno-miR-466b-1 | | |
| LOC679811 | 679811 | similar to RIKEN cDNA D930015E06 | XM_006224154 | rno-miR-181a-2 | | |
| Lemd3 | 680066 | LEM domain containing 3 | NM_001191000 | rno-miR-466b-1 | | |
| Mbnl2 | 680445 | muscleblind-like splicing regulator 2 | NM_001111064 | rno-miR-34b | | |
| Rbm41 | 680581 | RNA binding motif protein 41 | NM_001109420 | rno-miR-466b-1 | | |
| Qsox2 | 681023 | quiescin sulfhydryl oxidase 2 | NM_001109434 | rno-miR-1 | rno-miR-206 | |
| LOC681355 | 681355 | similar to potassium channel tetramerization domain containing 12b | XM_003752020 | rno-miR-466b-1 | | |
| LOC683430 | 683430 | similar to potassium channel tetramerization domain containing 12b | XM_001065910 | rno-miR-466b-1 | | |
| Eny2 | 685258 | enhancer of yellow 2 homolog (Drosophila) | NM_001130580 | rno-miR-466b-1 | | |
| Lrrtm2 | 685472 | leucine rich repeat transmembrane neuronal 2 | NM_001109469 | rno-miR-466b-1 | | |
| Nme4 | 685679 | NME/NM23 nucleoside diphosphate kinase 4 | NM_001109478 | rno-let-7a | | |
| | | | | | | |
| LOC687029 | 687029 | synovial sarcoma translocation gene on chromosome 18-like 2 | XM_003750606 | rno-miR-34b | | |
| LOC687808 | 687808 | solute carrier family 16, member 9 | XM_001080216 | rno-miR-466b-1 | | |
| Commd2 | 688478 | COMM domain containing 2 | NM_001109503 | rno-miR-466b-1 | | |
| Tslp | 688621 | thymic stromal lymphopoietin | XM_002725343 | rno-miR-1 | | |
| LOC688765 | 688765 | hypothetical protein LOC688765 | NM_001109512 | rno-miR-34b | | |
| Lin28b | 689054 | lin-28 homolog B | XM_006223929 | rno-let-7a | rno-miR-98 | |
| Cenpw | 689399 | centromere protein W | NM_001246319 | rno-miR-466b-1 | | |
| Ryr2 | 689560 | ryanodine receptor 2 | NM_001191043 | rno-miR-181a-2 | | |
| LOC689574 | 689574 | hypothetical protein LOC689574 | NM_001195503 | rno-miR-344b-1-3p | | |
| Srsf1 | 689890 | serine/arginine-rich splicing factor 1 | NM_001109552 | rno-miR-1 | rno-miR-206 | |
| Fam91a1 | 689997 | family with sequence similarity 91, member A1 | NM_001127578 | rno-miR-1 | rno-miR-206 | rno-miR-466b-1 |
| Zcchc3 | 690005 | zinc finger CCHC-type containing 3 | XM_002726251 | rno-let-7a | rno-miR-98 | |
| Fam168b | 690188 | family with sequence similarity 168, member B | NM_001271134 | rno-miR-1 | rno-miR-206 | |
| Ttll4 | 690512 | tubulin tyrosine ligase like 4 | XM_006245297 | rno-let-7a | rno-miR-98 | |
| Pou2af1 | 690528 | POU class 2 associating factor 1 | NM_001109599 | rno-miR-181a-2 | | |
| Katnbl1 | 691543 | katanin regulatory subunit B1 like 1 | NM_001109645 | rno-miR-466b-1 | | |
| Kctd4 | 691835 | potassium channel tetramerization domain containing 4 | NM_001109650 | rno-miR-466b-1 | | |
| Begain | 79146 | brain-enriched guanylate kinase-associated | NM_024163 | rno-let-7a | rno-miR-98 | |
| Slc6a1 | 79212 | solute carrier family 6 member 1 | NM_024371 | rno-let-7a | rno-miR-98 | |
| Gng5 | 79218 | G protein subunit gamma 5 | NM_024377 | rno-let-7a | rno-miR-98 | |

| | | | | | |
|---|---|---|---|---|---|
| Gk | 79223 | glycerol kinase | NM_024381 | rno-miR-466b-1 | |
| Hsd17b2 | 79243 | hydroxysteroid (17-beta) dehydrogenase 2 | NM_024391 | rno-let-7a | rno-miR-98 |
| Smad7 | 81516 | SMAD family member 7 | NM_030858 | rno-miR-344b-1-3p | |
| Ctbs | 81652 | chitobiase | NM_031023 | rno-let-7a | rno-miR-98 |
| Gatm | 81660 | glycine amidinotransferase | NM_031031 | rno-let-7a | rno-miR-98 |
| Gtf2b | 81673 | general transcription factor IIB | NM_031041 | rno-miR-466b-1 | |
| Itpr2 | 81678 | inositol 1,4,5-trisphosphate receptor, type 2 | NM_031046 | rno-miR-34b | |
| Vim | 81818 | vimentin | NM_031140 | rno-let-7a | |
| Slc20a1 | 81826 | solute carrier family 20 member 1 | NM_031148 | rno-let-7a | rno-miR-98 |
| Rab11a | 81830 | RAB11a, member RAS oncogene family | NM_031152 | rno-miR-466b-1 | |
| Cdh2 | 83501 | cadherin 2 | NM_031333 | rno-miR-466b-1 | |
| Pafah1b1 | 83572 | platelet-activating factor acetylhydrolase 1b, regulatory subunit 1 | NM_031763 | rno-miR-34b | |
| Smarcd2 | 83833 | SWI/SNF related, matrix associated, actin dependent regulator of chromatin, subfamily d, member 2 | NM_031983 | rno-miR-181a-2 | |
| Calb1 | 83839 | calbindin 1 | NM_031984 | rno-miR-34b | |
| Hsd17b12 | 84013 | hydroxysteroid (17-beta) dehydrogenase 12 | NM_032066 | rno-miR-466b-1 | |
| Col3a1 | 84032 | collagen, type III, alpha 1 | NM_032085 | rno-miR-1 | |
| Clcn3 | 84360 | chloride voltage-gated channel 3 | NM_053363 | rno-miR-1 | rno-miR-206 |
| Cdh11 | 84407 | cadherin 11 | NM_053392 | rno-miR-344b-1-3p | rno-miR-466b-1 |
| Arid4b | 84481 | AT-rich interaction domain 4B | NM_053421 | rno-miR-344b-1-3p | |
| Tec | 84492 | tec protein tyrosine kinase | NM_053432 | rno-miR-344b-1-3p | |
| Ppp1r9b | 84686 | protein phosphatase 1, regulatory subunit 9B | NM_053474 | rno-miR-466b-1 | |
| Gas7 | 85246 | growth arrest specific 7 | NM_053484 | rno-let-7a | rno-miR-98 |
| Slc44a1 | 85254 | solute carrier family 44 member 1 | NM_001033852 | rno-miR-1 | rno-miR-206 |
| Slc25a27 | 85262 | solute carrier family 25, member 27 | NM_053500 | rno-let-7a | rno-miR-98 |
| Ajuba | 85265 | ajuba LIM protein | NM_053503 | rno-miR-1 | rno-miR-206 |
| Rhoq | 85428 | ras homolog family member Q | NM_053522 | rno-miR-466b-1 | |
| Klf15 | 85497 | Kruppel-like factor 15 | NM_053536 | rno-miR-466b-1 | |

**Table S2**. Delta Ct (DCT) data showing relative Ct values compared to *miR-1*. MiRNAs having values >10 correspond to low abundance miRNA, while <6 denotes for high abundant miRNA. *DCt values were calculated as average Ct differences compared to average Ct values of *miR-1.*

| miRNA | Assay ID | Expression Rate (DCt)* |
|---|---|---|
| *rno-miR-344b-1-3p* | 464456_mat | n.d. |
| *rno-miR-466b-1-3p* | 002066 | 7.92 |
| *rno-miR-98-5p* | 000577 | 9.54 |
| *rno-let-7a-5p* | 000268 | 3.20 |
| *rno-miR-1-3p* | 464456_mat | 0.00 |
| *rno-miR-206-3p* | 000510 | 10.84 |
| *rno-miR-34b-3p* | 002618 | 8.31 |
| *rno-miR-181a-2-3p* | 464683_mat | 3.81 |

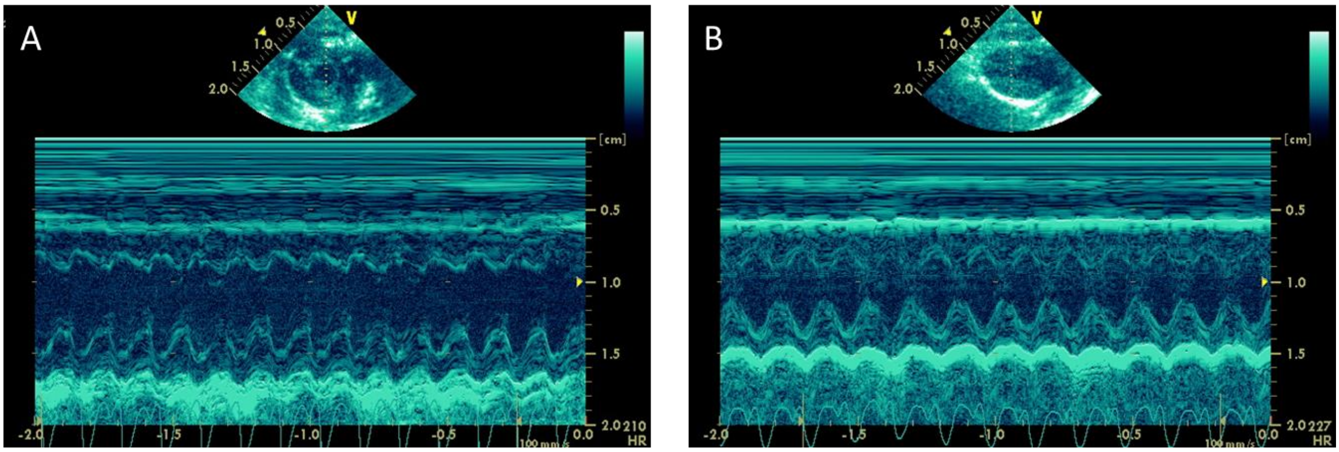


**Figure S1.** Representative ECG-gated M-mode images of transthoracic echocardiography. (**A**) Control and (**B**) sensory neuropathy. A reduction in left ventricular end-diastolic diameter in sensory neuropathic animals is seen. Echocardiography data are shown in Table S2.

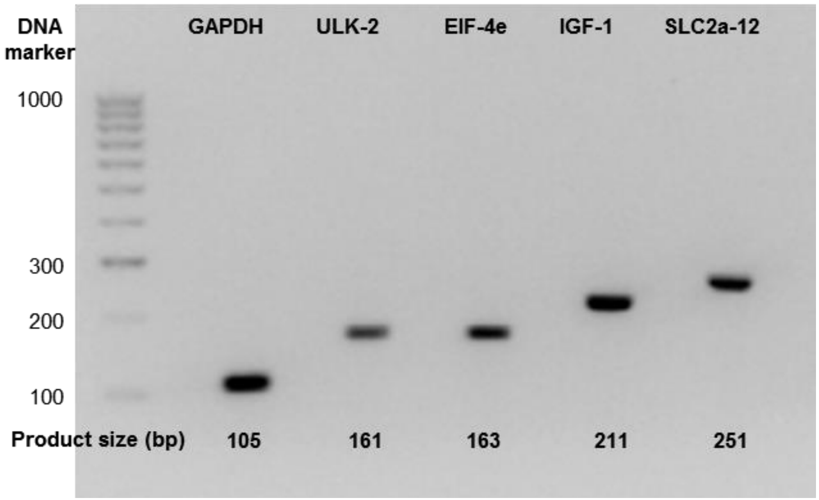


**Figure S2.** Amplified cDNA transcripts by the specific primers from pooled heart samples, separated by agarose gel ELFO. Bands show the transcripts separated by the size of the molecule. Lanes with no signals are negative controls (contain primers but no cDNA). DNA marker: HyperLadderTM IV molecular weight marker (100 bp–1013 bp; BIOLINE). *GAPDH*, glyceraldehyde-3-phosphate dehydrogenase; *ULK-2*, unc-51 like autophagy activating kinase 2; *EIF-4e*, eukaryotic translation initiation factor 4e; *IGF-1*, insulin-like growth factor 1; *SLC2a-12*, solute carrier family 2 facilitated glucose transporter member 12; and bp, base pairs.

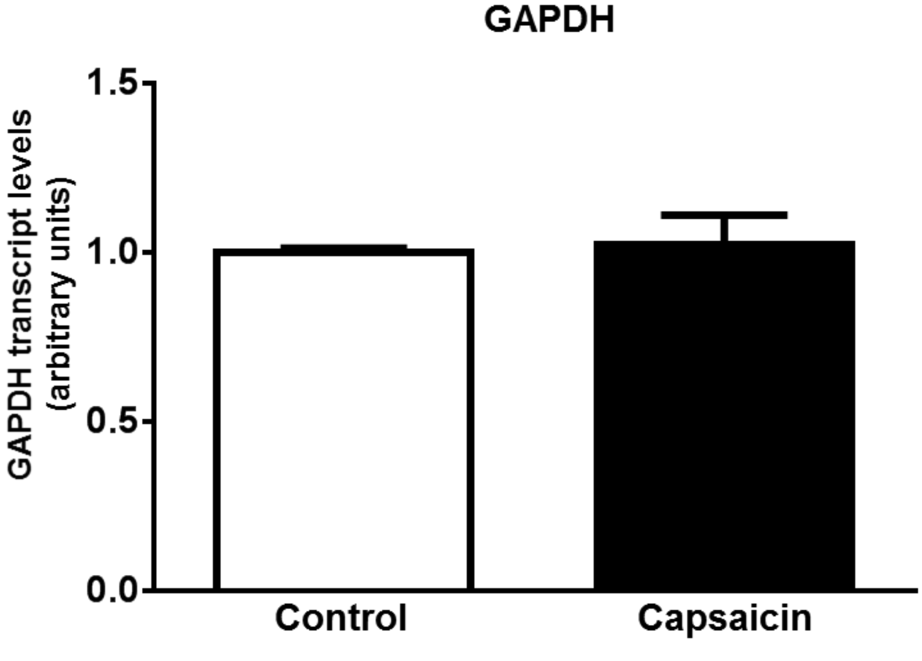

**Figure S3.** mRNA levels of GAPDH (glyceraldehyde-3-phosphate dehydrogenase) in capsaicin-treated rat heart samples as compared to vehicle controls. Data are expressed in arbitrary units as means ± S.E.M. ($n = 5–6$, $p = 0.79$; unpaired Student's t-test).
